# Transformation of Natural Genetic Variation into *Haemophilus Influenzae* Genomes

**DOI:** 10.1371/journal.ppat.1002151

**Published:** 2011-07-28

**Authors:** Joshua Chang Mell, Svetlana Shumilina, Ira M. Hall, Rosemary J. Redfield

**Affiliations:** 1 University of British Columbia, Department of Zoology, Vancouver, British Columbia, Canada; 2 University of Virginia School of Medicine, Department of Biochemistry and Molecular Genetics, Charlottesville, Virginia, United States of America; University of Toronto, Canada

## Abstract

Many bacteria are able to efficiently bind and take up double-stranded DNA fragments, and the resulting natural transformation shapes bacterial genomes, transmits antibiotic resistance, and allows escape from immune surveillance. The genomes of many competent pathogens show evidence of extensive historical recombination between lineages, but the actual recombination events have not been well characterized. We used DNA from a clinical isolate of *Haemophilus influenzae* to transform competent cells of a laboratory strain. To identify which of the ∼40,000 polymorphic differences had recombined into the genomes of four transformed clones, their genomes and their donor and recipient parents were deep sequenced to high coverage. Each clone was found to contain ∼1000 donor polymorphisms in 3–6 contiguous runs (8.1±4.5 kb in length) that collectively comprised ∼1–3% of each transformed chromosome. Seven donor-specific insertions and deletions were also acquired as parts of larger donor segments, but the presence of other structural variation flanking 12 of 32 recombination breakpoints suggested that these often disrupt the progress of recombination events. This is the first genome-wide analysis of chromosomes directly transformed with DNA from a divergent genotype, connecting experimental studies of transformation with the high levels of natural genetic variation found in isolates of the same species.

## Author Summary

The ability of bacteria to acquire genetic information from their relatives—called natural competence—poses a major health risk, since recombination between pathogenic bacterial lineages can help bacteria develop resistance to antibiotics and adapt to host defenses. In this study we transformed competent cells of the human pathogen *Haemophilus influenzae* with genomic DNA from a divergent clinical isolate and used deep sequencing to identify the recombination events in four transformed chromosomes. The results show that transformation of single competent cells is more extensive than expected, and suggests that transformation can be used as a tool to map traits that vary between clinical isolates.

## Introduction

For many bacteria, natural transformation is the dominant mode of genetic transfer between close relatives. These naturally competent bacterial species can actively take up DNA fragments from their surroundings and incorporate it into their chromosomes by homologous recombination [1]–[3]. Like sexual reproduction in eukaryotes, natural transformation moves alleles and loci between related bacterial lineages, allowing pathogens to share antibiotic resistances, antigenic determinants, and other virulence factors [4]–[10]. Population genetic studies have found evidence of pervasive recombination between lineages of human pathogenic bacteria, especially in taxa known to be naturally competent [11]–[12]. However such estimates of recombination are confounded by the other evolutionary forces of mutation and selection, and by the poorly understood demographic histories of the sampled isolates [13]–[15].

**Figure 1:**
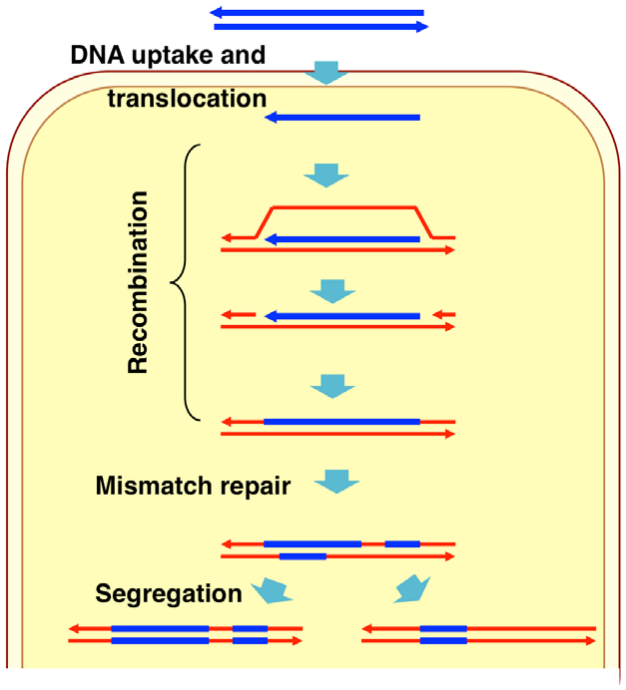
Model of natural transformation.

Naturally competent bacterial cells bind double-stranded DNA fragments at the cell surface but transport only single strands into the cytoplasm (Figure 1) [1]–[2]. Although several details of DNA uptake differ between Gram-positive and Gram-negative bacteria, in all bacteria the ensuing recombination between donor molecule and recipient chromosome is mediated by RecA homologs and other cytoplasmic proteins that limit DNA degradation and/or facilitate RecA-mediated strand exchange [1], [16]–[17]. In the laboratory competent cells can take up multiple long DNA fragments, although typically only a fraction of cells in a culture becomes competent [18]. As a consequence selection for transformation at one marker increases the fraction of cells found to be transformed by markers on independent DNA fragments.

During natural transformation, the extent to which incoming donor DNAs replace segments of recipient chromosomes is limited by the extent and type of sequence differences between the two, as is the case with other pathways that depend on homologous recombination [19]–[20]. Higher sequence identity between donor DNA and recipient chromosome increases transformation efficiency, while transformation by insertions and deletions is less efficient and requires flanking sequence homology [21]–[23]. The heteroduplex DNA created by strand exchange may be subsequently corrected by mismatch repair (to either a donor or recipient allele), or the uncorrected strands may segregate into daughter cells after DNA replication (Figure 1) [24]–[26].

The genomes of independent isolates of many bacterial species differ in two ways [27]–[30]. First, the 80–95% of two isolates' genome sequences that can be readily aligned differ at about 1–5% of bases. Second, the remaining 5–20% of unalignable DNA consists of structural variation resulting from past insertions, deletions, and more complex events. The finding that species show such high variation in gene content has led to a ‘supragenome hypothesis’, under which non-essential loci are frequently exchanged between lineages by transformation, potentially enabling rapid adaptation to varying conditions [29]–[31].

Natural genetic variation between bacterial strains has previously been used to characterize transformation at specific selected loci [18], [32]–[34], but no transformant has been genotyped across the entire chromosome. To investigate the factors that either promote or constrain the movement of genetic variation between otherwise clonal lineages of bacterial pathogens, we are using the well-characterized natural transformation system of *Haemophilus influenzae*, combining inexpensive sequencing technology with the availability of complete genome sequences of divergent strains. Here we report a high coverage sequence analysis of four *H. influenzae* genomes derived by natural transformation of a recipient strain with donor DNA of another strain differing at ∼40,000 genetic markers (∼2.5% of aligned positions and ∼300 indels and other rearrangements).

## Results

### Preliminary genetic analysis

**Figure 2:**
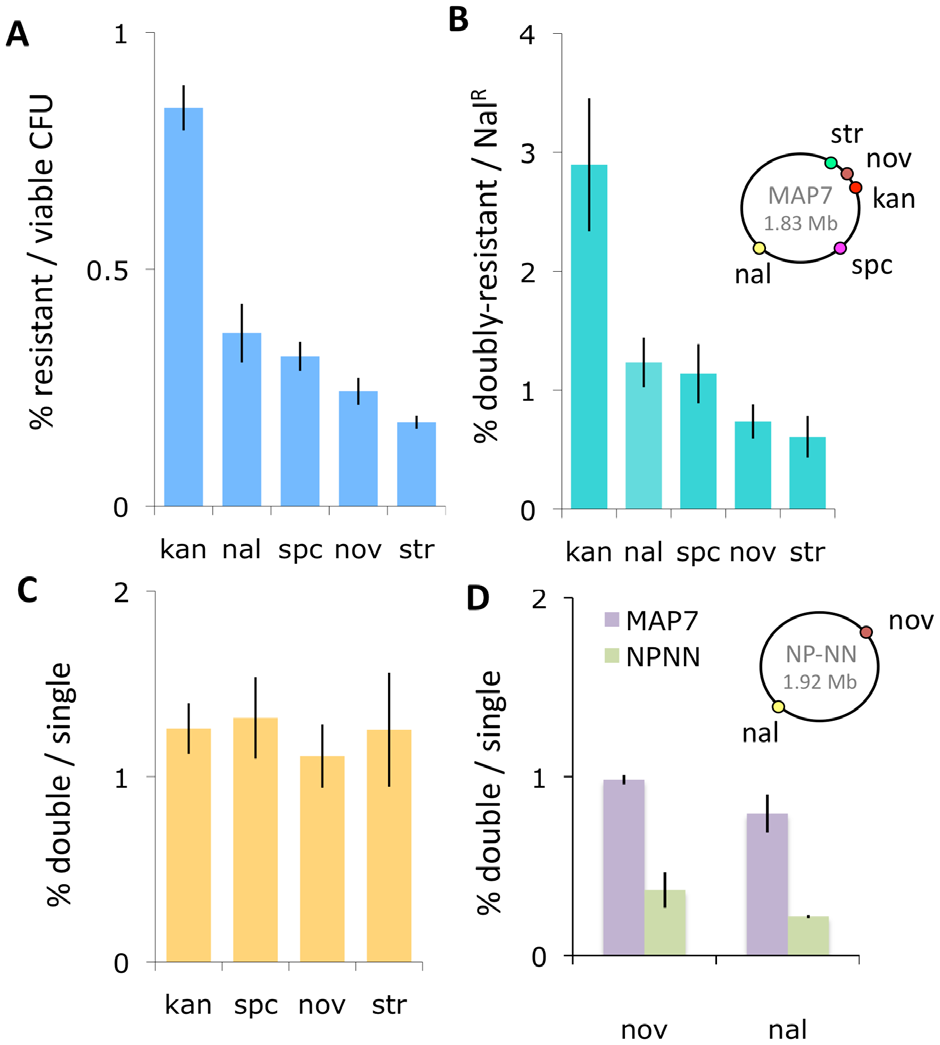
Natural transformation of H. influenzae. The inset chromosome maps show the positions of the antibiotic resistance markers in the two donor strains. (**A**) Transformants per viable cell at five markers using MAP7 donor DNA. (**B**) Double-selected transformants per single-selected transformant using MAP7 donor DNA. For Kan^R^, Spc^R^, Nov^R^, and Str^R^, the selection was for the distant Nal^R^ marker. For Nal^R^, the distant selection was at each of the other 4 markers. (**C**) Nal^R^ transformants per selected transformant using MAP7 donor. (**D**) Transformation of MAP7 vs. NP-NN donor DNA markers into Rd-RR per competent cell.

Competent cultures of *H. influenzae* are reported to contain many non-competent cells [18]. To avoid wastefully sequencing clones derived from non-competent cells, we chose for sequencing clones that had acquired a phenotypic marker in a standard transformation experiment. Before doing this, we confirmed that selection for transformation at one locus does not compromise transformation at distant loci, using donor DNA purified from the multiply marked Rd strain MAP7 [35] to transform our standard laboratory Rd strain Rd-RR (strains used are listed in Table 1). Figures 2A and B show that the relative transformation frequencies at five loci were not altered by selection for a distant marker, and Figure 2C shows that the transformation frequency of a nalidixic acid resistance marker (Nal^R^) did not vary when any of 4 distant markers is used for selection of transformed clones.

**Table 1:** Strains used.

Stock #	Name	Resistance Markers	Reference
RR722	Rd-RR	none	[38]
RR666	MAP7	Nov Nal Str Kan Spc Vio	[35]
RR1350	86-028NP	none	[39]
RR3131	NP-NN	Nov Nal	This work
RR3135	Nov1	Nov	This work
RR3136	Nov2	Nov	This work
RR3137	Nal1	Nal	This work
RR3138	Nal2	Nal	This work

Accurate mapping of recombination events depends on the density and distribution of sequence differences between the donor and recipient genomes. We chose the clinical isolate 86-028NP as the source of donor DNA because, like Rd, it has been completely sequenced and annotated, and because its genome differs from Rd at about 2.4% of their alignable bases, typical for a pair of *H. influenzae* isolates [30], [36]. To provide phenotypic markers in 86-028NP for transformant selection, we introduced Nov^R^ (novobiocin resistance) and Nal^R^ alleles from MAP7 by transformation with short PCR fragments.

We used this doubly marked strain (NP-NN) to investigate how strongly the sequence divergence between NP-NN and Rd-RR limits transformation. Figure 2D shows that the sequence divergence of the NP-NN donor DNA at or near the Nov^R^ and Nal^R^ alleles reduced transformation efficiency into recipient chromosomes by ∼3-fold, compared to Rd-derived MAP7 donor DNA.

### Genomic DNA sequencing of transformed clones and controls

To identify recombination events in clones transformed with chromosomal DNA from a divergent strain, we selected four Rd-RR clones that had been transformed with NP-NN chromosomal DNA to either a Nov^R^ (transformants Nov1 and Nov2) or a Nal^R^ (transformants Nal1 and Nal2) phenotype. Acquisition and processing of sequence data for these clones is described in detail in the Materials and Methods. Briefly, the Illumina GA2 sequencer [37] was used to obtain a high yield of short paired-end sequence reads from genomic DNA of these four transformants (two individually and all four as a pool), and also to individually resequence the genomes of the Rd-RR recipient and NP-NN donor strains as controls (Table 1 and Table S1). Each set of paired-end reads was separately aligned to each of the two reference genomes (Rd and 86-028NP [38]-[39]) using the alignment software BWA [40], and pileups and consensus base calls at each reference position were generated using the SamTools software package [41]. Since these genomes are <2 Mb, genome coverage per set of reads was high, with median read depths of ∼400 per mapped reference position (Table S2 and S3 and Figure S1).

For each set of reads, the base corresponding to each position in each of the two reference genomes was classified as: (a) the same as the reference, (b) different from the reference, (c) ambiguous, or (d) unmapped (summarized in Tables 2 and 3). Ambiguous positions were those where the specific base at a position could not be confidently identified, likely due to sequencing or read-mapping artifacts (see Materials and Methods). Positions were classed as unmapped if none of the reads aligned, either because the positions were absent from that DNA or unmapped for other reasons.

**Table 2:** Summary of read mapping to Rd (KW20).

	Rd-RR	NP-NN	Nov1	Nal1	Pool
% Matched a	99.7%	89.2%	99.7%	99.7%	95.9%
Variant b	277	39,049	1,441	1,530	262
Ambiguous c	4,459	10,353	4,411	3,675	74,374
Unmapped d	112	148,510	111	867	38

a Percent of bases that unambiguously matched their mapped reference base. The Rd (KW20) reference is 1,830,138 bp.

b Number of bases that unambiguously differed from their mapped reference base.

c Number of bases whose identity at mapped positions was ambiguous, defined where either the SamTools consensus caller identified a non-ACGT base, or because the frequency of non-reference variants at that position was between 0.05 and 0.95.

d Number of reference genome positions that were not mapped by the indicated set of sequence reads.

**Table 3:** Summary of read mapping to 86-028NP.

	Rd-RR	NP-NN	Nov1	Nal1	Pool
% Matched a	84.8%	99.8%	85.1%	85.0%	82.4%
Variant b	38,770	48	37,633	37,710	34,436
Ambiguous c	10,786	2,858	10,475	9,989	69,206
Unmapped d	241,237	22	236,977	239,666	234,173

a Percent of bases that unambiguously matched their mapped reference base. The 86-028NP references is 1,914,490 bp, respectively.

b Number of bases that unambiguously differed from their mapped reference base.

c Number of bases whose identity at mapped positions was ambiguous.

d Number of reference genome positions that were not mapped by the indicated set of sequence reads.

### Control analyses of donor and recipient re-sequencing data

**Figure 3:**
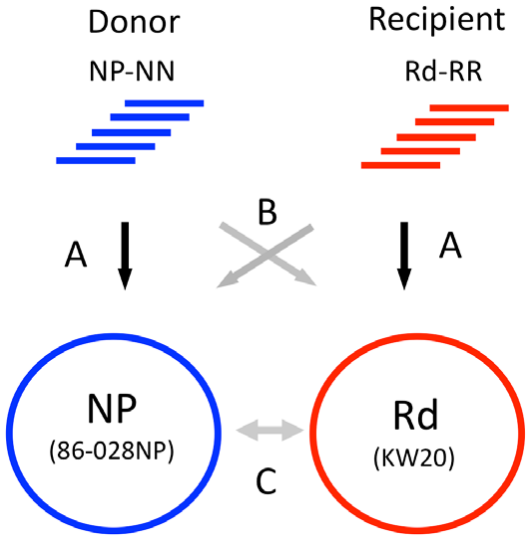
Control alignments. **Step A.** *Self-alignments:* Identifies intra-strain variants and ambiguous positions. **Step B.** *Reciprocal alignments:* Identifies putative SNVs and indels between donor and recipient. **Step C.** *Reference alignments:* Whole-genome alignment of the 86-028NP and Rd reference sequences identifies a set of SNVs for cross-validation with the self- and reciprocal-alignments.

Before transformant data sets were analyzed to identify recombination events, the control sequence reads were used to (1) identify differences between the published reference genome sequences and the genomes of the donor and recipient strains we used; (2) confirm the reliability of single-nucleotide variants (SNVs) for distinguishing between donor and recipient sequences; and (3) identify positions that were systematically error-prone, ambiguous, or unmapped in the alignment of reads to references. The three steps (A, B and C) of these control analyses are illustrated in Figure 3.

#### Step A. Self-alignment of donor and recipient reads to their own references

Aligning the sequence reads from the recipient and donor strains to their corresponding reference genomes provided two controls. First, it showed that our sequencing approach was comprehensive and accurate: at >98% of the positions the aligned reads agreed with the reference with <1% error (Tables 2 and 3, and Tables S4 and S5). Although the Rd-RR reads disagreed with the Rd reference at several hundred positions, many of these appear to be errors in the 1995 reference sequence. Second, these self-alignments allowed other potential artifacts to be accounted for by identifying (i) sequence differences between the strain and their respective references (Table 4), (ii) several thousand positions where sequencing results were ambiguous (Figure S2), and (iii) a small number of unmapped positions at apparent deletions (Tables 2 and 3).

**Table 4:** Correction for variants detected by self-alignment (Steps A and C).

Reference SNVs a	42156
Rd alleles in NP-NN b	−42
Rd-exclusive alleles c	−237
Solved ambiguous Rd d	−92
NP-NN-specific e	+3
MAP7-specific in NP-NN f	+3
Rd-RR specific g	+40
**Adjusted validation set** h	**41821**

a SNVs detected by Mauve whole-genome alignment of the reference Rd and 86-028NP genomes.

b Alleles shared between Rd-RR and NP-NN, due to introduction of MAP7 antibiotic resistance alleles.

c Alleles shared between Rd-RR and NP-NN that are variant only in the Rd reference.

d Alleles shared that were originally non-ACGT bases in the Rd reference.

e Alleles found only in NP-NN.

f Alleles in NP-NN that are MAP7-specific.

g Alleles found only in Rd-RR.

h Final set of SNVs used for cross-validation (Table 5).

**Table 5:** Cross-validation of SNVs detected by reciprocal alignment (Steps B and C).

	Rd-RR to NP	NP-NN to Rd
Adjusted validation set a	41,821	
Detected variants b	38,770	39,049
Validated variants c	37,915	38,048
False negatives d	3,634	3,711
Ambiguous e	(1537)	(1538)
Unmapped f	(2097)	(2173)
False positives g	809	955
**Cross-validated** h	**37,201 (88.9%)**	

a SNVs found by whole-genome alignment after correcting for Step A (Table 4).

b Unambiguous SNVs detected by reciprocal alignment.

c SNV detected by reciprocal alignment, and also found by whole-genome alignment.

d SNV not detected by reciprocal alignment, but found by whole-genome alignment.

e False negative due to SNV having an ambiguous base assignment.

f False negative due to SNV missing from the reciprocal alignment.

g SNV detected by reciprocal alignment, but not found by whole-genome alignment.

h SNV detected by both reciprocal alignments and also whole-genome alignment (total % cross-validation in parentheses).

#### Step B. Reciprocal alignment of donor and recipient reads

We next evaluated the alignment of donor and recipient reads to the alternative reference genomes (Rd-RR aligned to 86-028NP, and NP-NN aligned to Rd; Tables 2 and 3). These alignments served to identify variant, ambiguous and unmapped positions arising when reads are aligned to a reference with substantial sequence divergence. The ∼40,000 SNVs detected by the two reciprocal alignments were roughly consistent with the whole-genome alignment of the Rd and 86-028NP reference sequences described below (Tables 2 and 3, and Tables S6–S8).

Many of the positions identified as ambiguous in the self-alignments were also ambiguous in the reciprocal-alignments (44% and 58% overlap), suggesting that these positions suffer from systematic and persistent sequencing or mapping artifacts (example in Figure S3A). However, more than twice as many positions were classified as ambiguous in reciprocal alignments than in self-alignments (Tables 2 and 3, and Figure S2). Many of these were due to sequence reads that consistently misaligned at regions of high divergence between donor and recipient (example in Figure S3B), causing lower read depths and higher variant frequencies at these positions than in self-alignments (Figure S4).

Because of the substantial number of indels and other rearrangements between donor and recipient (Table S8), many reference positions were not mapped by reciprocal alignment (Tables 2 and 3, and Figure S5). 12.6% of 86-028NP positions had no recipient reads mapped, and 8.1% of Rd positions had no donor reads mapped. Because of the high sequence coverage of these genomes, these unmapped positions served as markers to identify structural variation between the two (see below).

#### Step C. Cross-validation of SNVs between donor and recipient

Most of the mapped SNVs found by reciprocal alignment were expected to identify genuine polymorphisms, but others were mistakes generated by read mapping artifacts. To independently validate SNVs, we aligned the two reference genomes using the Mauve whole-genome alignment software [42] (Tables S6–S8). After correcting for the variation detected between the strains and their references (Step A, Table 4), we used this set of SNVs to cross-validate the sequence variants identified by the reciprocal alignments (Step B). This gave a final set of 37,201 positions for which the same distinct variants were detected in both the alignments of control reads to their reciprocal references (Step B), and the alignments of the two references to each other (Step C). The positions of these cross-validated SNVs were used to identify the donor- and recipient-specific alleles in transformed clones (see below); they captured 88.9% of the total SNVs identified by whole-genome alignment, with a mean spacing of 52 bp (median 15 bp). Exclusion of SNVs that failed this cross-validation test modestly reduced the number of markers available for recombination analysis, but it eliminated artifacts that would otherwise have been interpreted as novel alleles (new mutations) and as interruptions of recombination tracts (Table 5).

### Recombination tracts in two individually sequenced transformed chromosomes

**Figure 4:**
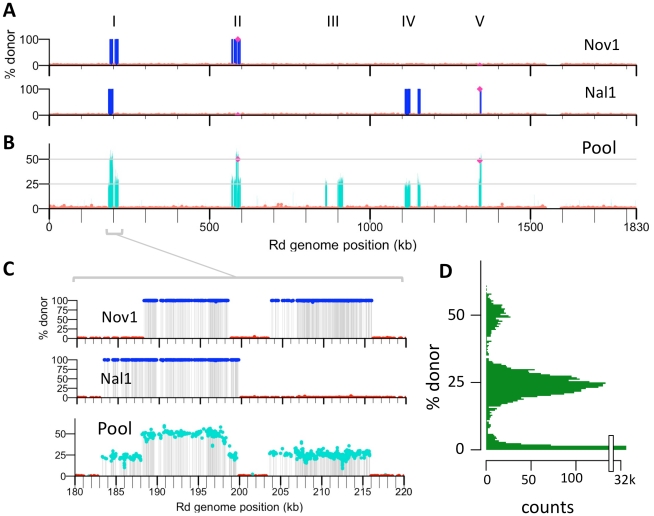
Identifying transformation events genome-wide. (**A**) Donor allele frequency at cross-validated SNVs in the Nov1 and Nal1 transformants plotted against recipient genome position. Donor SNVs are blue and recipient SNVs are red. Roman numerals indicate intervals where transformation was detected in either **A** or **B**. The selected Nov^R^ and Nal^R^ alleles in intervals II and V are purple. (**B**) Donor allele frequency at cross-validated SNV positions in the pool of four transformants (Nov1, Nov2, Nal1, and Nal2) plotted against recipient genome position. Coloring as in **A**, with turquoise used for intermediate donor allele frequencies. (**C**) Expanded view of interval I, as in **A** and **B**. All five intervals are shown in Figure S7. (**D**) Histogram of the donor allele frequency at cross-validated SNV positions in the pool of four transformants mapped to the Rd reference.

**Figure 5:**
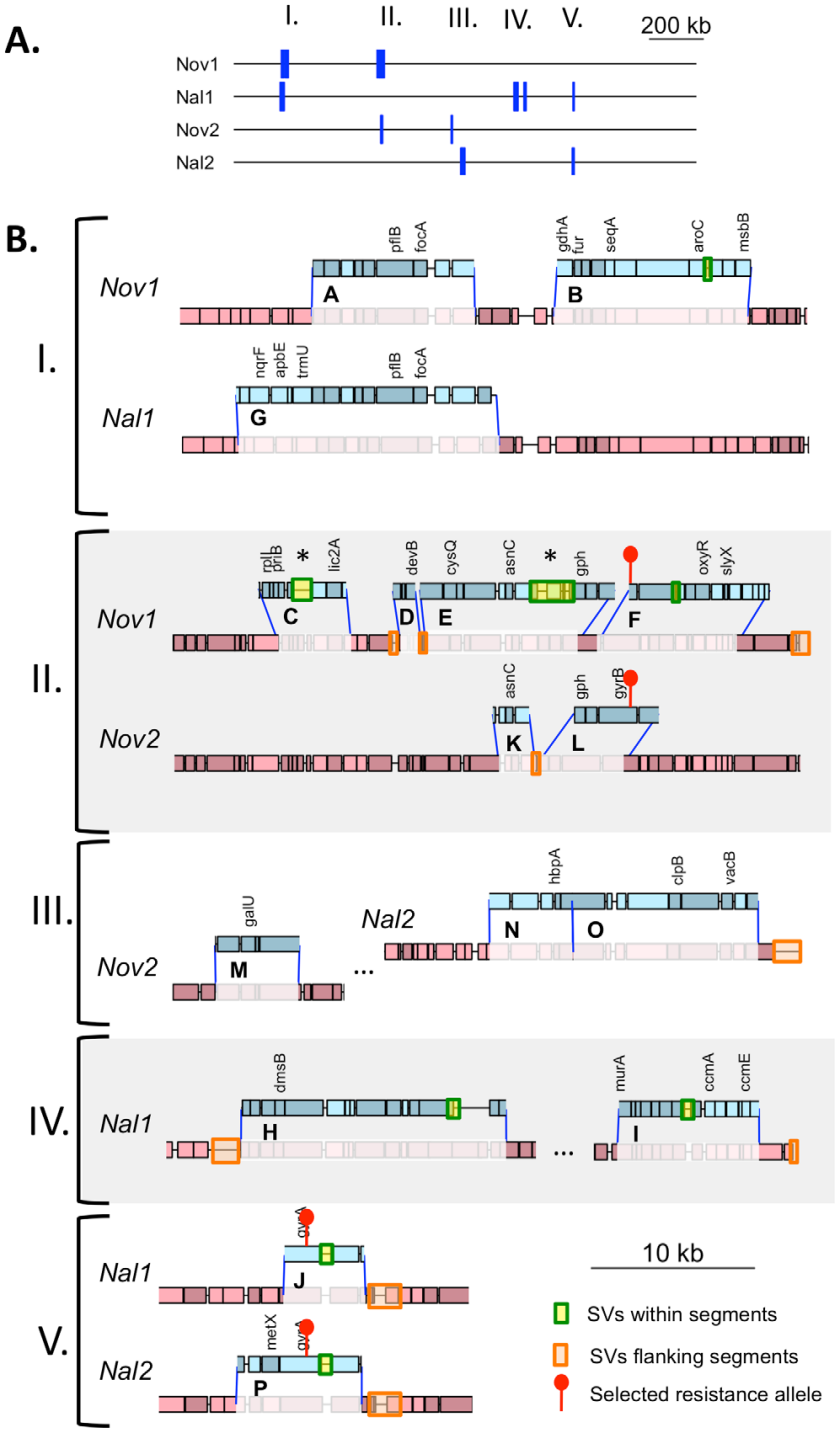
Recombination events detected in four transformants. (**A**) Summary of donor segments in each transformant are illustrated as blue bars. (**B**) Detailed illustrations of recombination tracts in each interval showing gene annotations. Donor segments are shown in blue, and recipient sequences are shown in red. Pale shading indicates genes on the plus strand and dark shading indicates genes on the minus strand. Dark blue lines joining donor and recipient segments indicate recombination breakpoints. The key in the lower right shows additional annotation of the selected antibiotic resistance alleles and the locations of structural variation between the genomes. Exact donor segment breakpoints and additional data on each segment are provided in Table S9 and Table S10 for the Rd and 86-028NP genome coordinates, respectively. Scale bars are shown for both (**A**) and (**B**).

To determine the locations of donor alleles in transformant clones Nov1 and Nal1, sequence reads were aligned to both reference genomes and each cross-validated SNV position was classified as donor-specific, recipient-specific, or ambiguous. The two clones contained 1,133 and 1,213 donor-specific SNVs, while nearly all of the remaining cross-validated positions contained recipient-specific SNVs (Figure 4A and Table 6). As expected for the products of homologous recombination, donor-specific alleles in the transformed clones were found in contiguous runs, which we term donor segments (Figure 4A, Figure 5, and Figure S7).

**Table 6:** Summary of transforming donor DNA in 4 recombinants.

	Donor segment size				Donor-specific SNVs		
Clone	# a	length b	% total c	% selected d	# e	% total f	% selected g
Nov1	6 (2)	46,199	2.52	51.6	1,133	3.01	47.9
Nal1	4 (4)	46,690	2.55	10.7	1,218	3.27	8.9
Nov2	3 (2)	12,712	0.70	59.0	208	0.56	63.0
Nal2	3 (2)	24,686	1.35	31.4	624	1.68	23.7

a Total number of donor segments. Number in parentheses indicates the total number of recombination tracts, or clusters of donor segments separated by <10 kb.

b Combined length of donor segments, measured as the sum of distances between the outermost donor-specific SNVs.

c Percent of recipient genome length replaced by donor segments (out of 1,830,138 bp).

d Percent of donor segment lengths found clustered at the selected Nov^R^ or Nal^R^ alleles.

e Total number of cross-validated donor-specific SNVs.

f Percent of total cross-validated SNVs (out of 37,201 cross-validated SNPs).

g Percent of donor-specific SNVs in segments clustered at the selected sites.

The lengths of the 10 donor segments in the Nov1 and Nal1 transformants ranged from 1.2 kb to 16.6 kb (Figure S5 and Figure S6). As expected, transformant Nov1 had a donor segment spanning the Nov^R^ allele at *gyrB* (Segment F), and transformant Nal1 had a donor segment spanning the Nal^R^ allele at *gyrA* (Segment L). Nov1 contained five additional donor segments, two separated by only 4.5 kb and the other three adjacent to the selected Segment F. Nal1 contained three other widely spaced segments in addition to the selected Segment L; one of these overlapped one of those in the Nov1 transformant by 10.9 kb (Segments A and G, shown expanded in Figure 4C and Figure 5). Additional analysis of these segments is presented below.

### Recombination tracts in a pool of four transformed chromosomes

The ∼400-fold coverage obtained per sequencing lane was much higher than needed, so we tested whether sequencing a pool of genomic DNA from four transformants could accurately identify donor segments without compromising the resolution of recombination breakpoints. Equal amounts of four genomic DNAs were pooled and sequenced (two transformant clones, Nov2 and Nal2, and as internal controls, the individually sequenced clones Nov1 and Nal1). The reads from this pool were aligned to the donor and recipient references, and the allele frequencies at each position for each reference were calculated (Tables 2 and 3). In this analysis donor alleles acquired by transformation of one clone will be given ‘ambiguous’ base assignments, with donor alleles at 25%. These are seen in the allele-frequency histogram in Figure 4D as the large peak centered on 25%; the smaller peak centered on 50% reflects the donor alleles present in two clones.

When plotted against chromosome coordinate, the recombination breakpoints of the donor segments in the pool are evident as abrupt transitions of donor-allele frequency (Figure 4 and Figure S7). For this pool of 4 genomes, donor segments are seen as intervals of contiguous ∼25% or 50% donor allele frequency. As expected, overlapping segments were seen at the selected Nov^R^ and Nal^R^ alleles, with the Nov^R^ allele in the Nov1 and Nov2 transformants and the Nal^R^ allele in the Nal1 and Nal2 transformants (purple diamonds in Figures 4 and Figure S7). The previously identified overlapping segments A and G were also detected (in Nov1 and Nal1, respectively). The pool contained three more unselected donor segments specific to either Nov2 or Nal2. Allele-specific PCR was performed to determine which of the two clones these three donor segments were found; Segment M was in Nov2, while Segments N and O were in Nal2.

While the pooling approach was successful at precisely identifying recombination breakpoints and overlaps between donor segments in the four different clones, the assignment of endpoints to overlapping donor segments and to particular recombinant clones required additional information. The increasing availability and decreasing cost of multiplexed sequencing methods will partially circumvent this problem in the future.

### Properties of donor segments

In total, we identified 16 donor segments across the four transformants, spanning a total of ∼130 kb and containing 3,183 donor-specific SNVs (Table 6 and Tables S9 and S10). This is 7.1% of the Rd genome, or 6.0% if overlaps are only counted once. The 16 donor segments had a mean length of 8.1±4.5 kb, suggesting that transformation of very short DNA fragments is rare (at least with the high molecular weight donor DNA prep we used). The average amount of sequence introduced into each transformed clone (∼1.8%) was consistent with the transformation frequencies of individual selectable markers shown in Figure 2.

**Figure 6:**
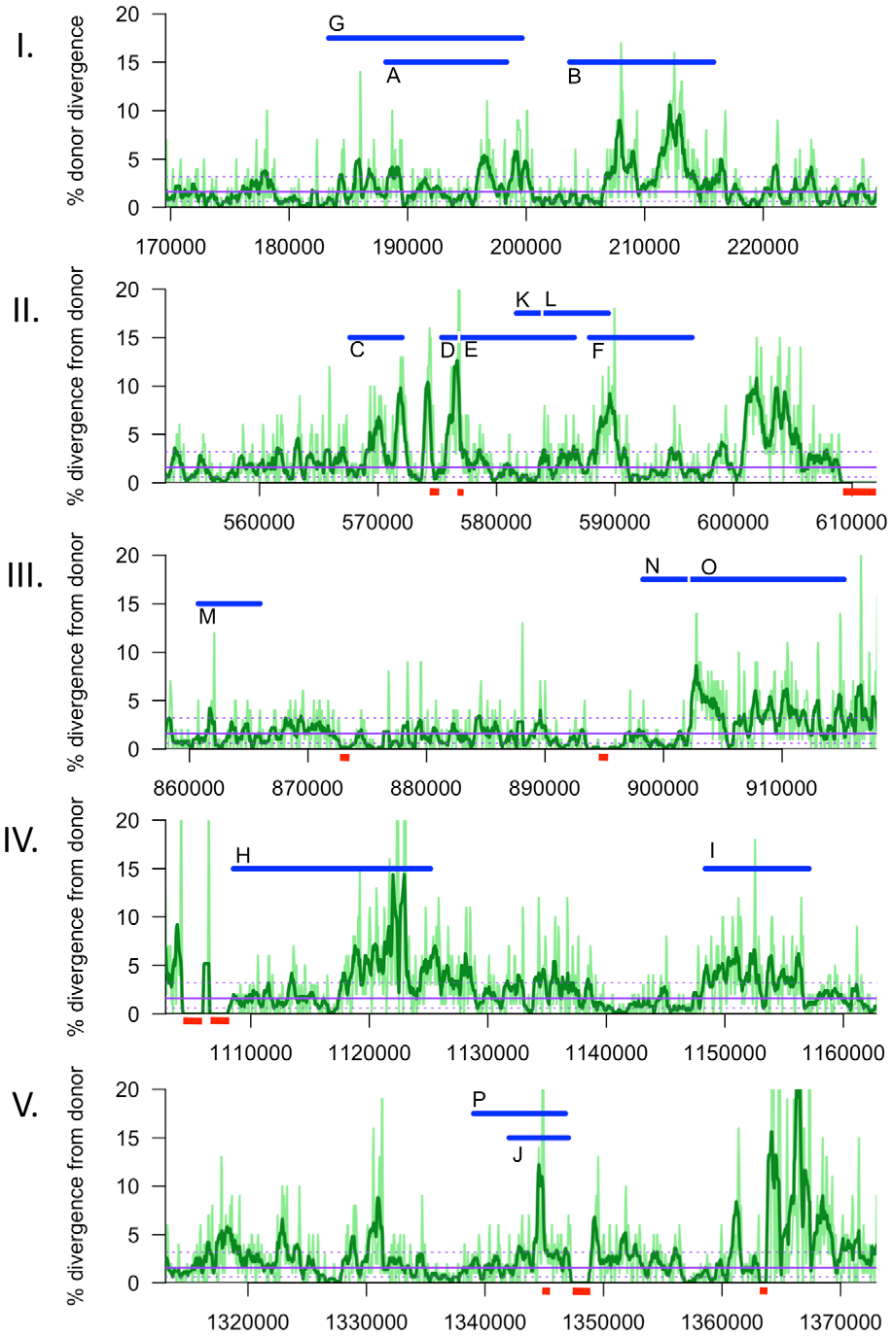
Sliding window analysis of % nucleotide divergence across the five intervals containing recombination tracts, I to V. Donor segments are shown as horizontal blue bars. Dark and light green lines indicate 1 kb and 250 bp window sizes, respectively (both with a step size of 100 bp). The solid purple line shows the median genome-wide divergence, and the dotted purple lines shows the 25% and 75% quartiles. Red bars below the axis indicate positions of large recipient-specific sequences (deletions in the donor), where the % divergence is artificially reduced to 0%. The spacing between Segments D/E, K/L, and N/O are not to scale, so that the position of putative restoration repair are clearly visible.

Although transformation might be expected to preferentially occur at regions with low sequence divergence, the regions participating in recombination had divergences typical of the whole genome (2.4±0.9% vs 2.3%). A more detailed analysis of sequence divergence in these regions is shown in Figure 6 Notably, the extent of sequence divergence is locally highly variable, ranging from less than 1% to more than 15% within only a few kilobases. However this variation did not appear to affect recombination, since all donor segments contained regions of both high and low divergence, and there were no obvious correlations between recombination breakpoints and extremes of divergence. Notably, recombination was not interrupted even when divergence was as high as 20% (light green line, 250 bp sliding windows).

The adjacent locations of many donor segments (Figure 5) likely resulted from disruption of longer transforming DNA fragments rather than independent events. For example the 6 donor segments in Nov1 were found in 2 clusters of 22 and 24 kb. Across all four clones, there were 6 instances of apparent disruptions within longer “recombination tracts”, where adjacent donor segments were separated by relatively short intervals (<10 kb) of recipient-specific alleles. The longest is the 5.3 kb interval separating segments A and B in transformant Nov1, and the shortest is the single recipient SNV dividing Segments N and O in transformant Nal2 (Tables S9 and S10). When the 16 donor segments were treated as 10 clusters, the mean recombination tract length was 14.2±8.8 kb.

**Figure 7:**
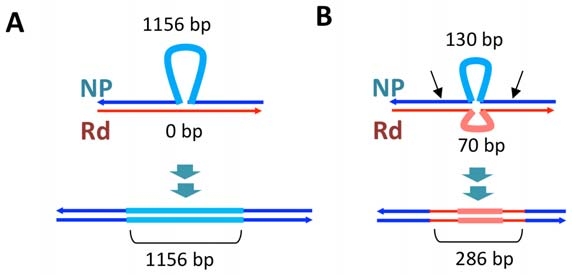
Transformation at structural variation. The top drawings illustrate the inferred joint molecule intermediates that yielded the recombination products illustrated in the bottom drawings. In (**A**) the 1.2 kb insertion in Segment C is shown, and in (**B**) the putative restoration repair at an insertional deletion disrupting Segments D and E is shown. Thin dark lines show aligned sequence (blue and red for donor and recipient sequences, respectively). Thick pale lines show unaligned indel differences between the genomes. For **B**, black arrows show putative cut sites by a mismatch correction endonuclease.

Recombination not only brought thousands of donor-specific SNVs into the transformant genomes but introduced several donor-specific insertions and deletions (Table S11) resulting in some donor segments being different lengths than the recipient segments they replaced (Figure 5, Tables S9 and S10). In particular, strain Nov1 received two large donor-specific insertions (1.2 and 2.7 kb) as parts of Segments C and E (Figure 5 asterisks and Figure 7A). These were confirmed by read depth analysis along the two reference sequences (Figure S8).

On the other hand, indels and other structural variants between the donor and recipient chromosomes appear to have blocked progression of strand exchange in several instances (Figure 5 and Table S12). Of the 32 donor segment breakpoints, 12 are within 5 kb of indel or other structural variation; 6 of these are within 3 of the 6 apparent disruptions described above and thus are likely sites of restoration repair. Indeed, one structural variant gave different outcomes in different recombinants: the 2.7 kb donor insertion allele that was acquired by strain Nov1 was not inserted into strain Nov2, but instead a short segment of recipient sequence interrupts donor segments K and L. Figure 7B illustrates another example of putative restoration repair at an insertional deletion difference between the donor and recipient, as indicated by the interruption between Segments D and E by the recipient insertion allele along with 26 flanking recipient SNVs (Figure S8).

## Discussion

The plummeting cost of deep sequencing allowed us to characterize the genome-wide consequences of natural transformation, but the ability of this analysis to account for artefacts depended on our high-coverage control sequencing of the donor and recipient genomes. Aligning these control reads to the two reference genome sequences revealed many positions prone to ambiguity or false-positive SNV calls. In the absence of these controls, such artifacts would have mistakenly been interpreted as recombination-induced mutations, since mapping reads to divergent references generated these erroneous variants, while mapping reads to highly similar references did not. The frequency of these artifacts depends not only on nucleotide divergence, but also on the spectrum of structural variation and the complexity of the genome. Analysis of such high-coverage control datasets will be essential for reference-guided assembly approaches that use data with lower coverage, such as that obtained using inexpensive multiplexing methods.

*H. influenzae's* normal environment is the mucosal layer of the human respiratory tract, which contains abundant DNA, much of it high molecular weight like that we used [43]. The broad spectrum of sequence differences between donor and recipient used in these experiments is typical of the natural genetic variation between *H. influenzae* strains and present in the human host [44]-[45]. However most of the DNA in respiratory mucosa is from human cells and, although bacterial DNA is known to be abundant in biofilms, its fragment sizes and composition in mucus are not known. The short DNA fragments also present in mucus may be taken up more efficiently than long fragments, since *H. influenzae* cells take up more fragments when fragments are short (40 fragments of 120 bp) [46]–[47], but the implications for transformation are not clear. Competent cells incubated with short donor DNAs might acquire more donor segments, but short fragments will also be more severely affected by the exonucleolytic degradation that accompanies translocation into the cytoplasm. *H. influenzae'*s preference for DNA containing uptake sequences (see below) will also affect both the sources and sizes of the fragments cells take up.

Analysis of recombination tracts showed that the four transformants had replaced ∼1–3% of their genomes with 3–6 segments of donor DNA ranging in length from 1.2 to 16.6 kb. The number of donor segments per transformant agrees well with the ∼3.3 fragments found to be taken up per cell in laboratory experiments using long 14.4 kb fragments [47]. The lengths of the donor segments we found in *H. influenzae* are similar to those reported from analysis of naturally occurring recombination tracts observed in *Neisseria meningitidis* at specific loci[48], but contrast with the shorter tracts seen for *Helicobacter pylori* (∼0.5–3.5 kb) in experiments using DNAs of similar divergence to ours [33]–[34], [49]. The difference suggests that population genetic models for measuring recombination in nature will require incorporating species-specific estimates of the distribution of recombination tract lengths [15].

The lengths of donor segment found in recombinant chromosomes may underestimate the original lengths of DNA fragments participating in uptake and recombination, because clustering of donor segments suggests that longer incoming DNA fragments are often disrupted before transformation is complete. Similar clustering of donor segments was seen when recombination at a single locus was examined in *Helicobacter pylori* [33]–[34]. The clustering of *H. influenzae* donor segments is unlikely to be due to chance, because of the small number of donor segments in each transformant. More probable explanations are that (1) cytosolic or translocation endonucleases degrade incoming DNAs prior to strand exchange, or (2) sequence heterology blocks progression of strand exchange, with the heterologous sequences trimmed away by nucleases.

Intracellular cleavage of incoming DNA by restriction enzymes has been proposed for competent *Helicobacter pylori* [49], but this is problematic because, in both *H. pylori* and *H. influenzae,* only single DNA strands are thought to enter the cytoplasm [50]–[51]. Although McKane and Milkman have shown that restriction can create clustered recombination tracts in *E. coli* transduction experiments [52], the single strands brought into the cytoplasm by transformation are not normally substrates for restriction enzymes, and donor strands recombined into the chromosome will be protected from restriction by the methylation of the base-paired recipient strands. The effect of restriction in *H. pylori* may instead be due to the accumulation of extracellular restriction enzymes during the long transformation protocol [34]. Similar accumulation might be a transformation-limiting factor for many species that normally live in mixed-species biofilms, whenever environmental DNA encounters restriction enzymes derived from other strains or species.

We found no evidence that recombination preferentially occurred in regions of lower nucleotide divergence than the genome-wide average. Instead, sequence divergence varied on a scale much shorter than the donor segments, with most segments spanning local regions of both high and low divergence (Figure 6). Although strand exchange of short fragments is known to be dramatically inhibited by sequence divergence of >10% [19]–[20], most donor segments contained one or more regions with >15% divergence. This suggests that, although strand exchange may initiate between regions of high sequence identity, it readily extends into and through regions with many mismatches. Measuring the effect of divergence on recombination break points and interruptions will require sequencing many more recombinants.

Effects of structural variation on recombination were evident even with this small sample size, as heterologous sequences were much more common at donor-segment breakpoints than expected from their abundance in the recombining genomes, *e.g.* between the clustered segments C and D, D and E, and K and L (Figure 5 and 6B). This is consistent with previous genetic experiments showing that insertions and deletions transform at much lower rates than do substitutions [22] and may be due to inhibition of strand exchange or to subsequent excision of heteroduplex from recombination intermediates by a mismatch correction mechanism. However, at other sites the donor versions of structural variation were acquired as parts of longer donor segments, showing that such accessory loci can indeed readily move by natural transformation.

Other factors could have influenced the transformation events we observed: (1) *H. influenzae*'s strong preference for DNA fragments containing uptake signal sequences (USS) biases transformation to USS-containing fragments [53]–[54]. However USSs are unlikely to have been a factor in these experiments, since they occur at a high density in the genome (∼1/kb) and the donor DNA fragments used typically contained dozens of USSs. (2) Segregation of uncorrected heteroduplex at the first post-transformation cell division could cause the extent of strand replacement in individual competent cells to be underestimated by up to 2-fold (Figure 1). We do not know the extent of heteroduplex correction at these or other independently transforming sites, nor how recombination tracts are distributed between the two strands of the originally transformed chromosome. Although the relatively short putative restoration repair events observed in this study might suggest that heteroduplex correction only act on parts of larger heteroduplex recombination products, other repair events might have completely removed shorter segments of donor DNA.

Because clones chosen for sequencing had acquired one of two antibiotic resistance alleles from the donor, we were able to examine overlapping recombination events at each of these loci, detecting striking differences at the Nov^R^ locus. The selection for Nov^R^ and Nal^R^ also showed that unselected events are common, as 58% of donor alleles were found in segments distant from the selected loci. On the other hand, the 11 kb overlap between the unselected donor segments A and G was unexpected given the transformation frequencies of single markers (Figures 4C and 5), and a sufficiently large dataset might identify a transformation hotspot, as has recently been found in *Neisseria meningitidis* [55]. The overlapping sequences do not have any obvious distinguishing features: divergence between donor and recipient is typical, no virulence genes have been annotated, and density of USSs is slightly lower than the genome average.

In addition to the selected antibiotic resistance alleles, the recombination events characterized here had the potential to significantly change the cell's biology, both by introducing new genes and by creating new genetic combinations by homologous recombination both between and within genes alleles. In particular, the Segment E insertion contains four donor-specific ORFs, one encoding a predicted transposase, and the Segment C insertion contains the LPS biosynthesis gene *lic2C* (between *infA* and *ksgA*). Each recombinant clone also acquired donor-specific versions of 20–50 shared genes, and these may have altered phenotype both directly and because of new interactions with recipient alleles at unrecombined loci. Recombination breakpoints that were not at structural variation usually fell within genes (Figure 5) and, because of the high level of sequence variation, these are likely to have created novel recombinant alleles potentially with substantial changes to function.

The results presented above considered only four recombinant clones, but continuing advances in DNA sequencing technology and bioinformatics methods will allow characterization of many more recombinants under a variety of experimental conditions and using different donor DNAs. This will help bridge experimental studies of transformation with the population genomic approaches used to detect recombination between bacterial lineages in nature. The comprehensive identification of donor segments in a large set of experimentally transformed clones will also provide a novel resource for the genetic mapping of phenotypes that differ between the donor and recipient strains, such as their dramatic natural variation in transformability [56], as well as natural variation in pathogenesis-related traits like serum-resistance [57]–[58].

## Materials and Methods

### Culture conditions, competent cell preparations, and DNA purification

Standard protocols were used for growth and manipulation of *H. influenzae*, preparation and storage of competent cultures, and purification of high molecular weight chromosomal DNA from overnight cultures [35], [59]. Briefly, cells were grown in the rich medium sBHI and made competent by transfer of log-phase cultures to the starvation medium M-IV for 100 minutes before transformation experiments or storage in 15% glycerol at −80°C.

### Strains (Table 1)

The *H. influenzae* recipient strain Rd-RR (RR722) was obtained from H. O. Smith in 1988, and is separated by ∼10 passages (∼500 generations) from the KW20 Rd strain sequenced in 1995 [38] (NCBI *Taxonomy ID:* 71421). The donor strain NP-NN (RR3131; resistant to novobiocin and nalidixic acid (Nov^R^ and Nal^R^)) was derived from the clinical isolate 86-028NP [39] (RR1350, gift of Richard Moxon in 2006, NCBI *Taxonomy ID:* 281310); it is separated from the sequenced 86-028NP strain by ∼5 passages (∼250 generations). NP-NN was constructed by PCR-mediated transformation of 86-028NP with Nov^R^ and Nal^R^ amplicons of *gyrB* and *gyrA*, respectively, (both caused by point mutations). For the Nov^R^ allele of *gyrB*, a 2.6 kb fragment (Rd coordinates 585,533 to 588,096 bp) was amplified from MAP7 (RR666) chromosomal DNA. For the Nal^R^ allele of *gyrA*, a 2.8 kb fragment (Rd coordinates 1,341,635 to 1,344,397) was amplified.

### Transformation experiments

Transformation experiments used 2 µg of chromosomal DNA per 1 ml of M-IV competent culture (∼10^9^ cells) for a final DNA concentration of ∼1 genome equivalent per cell. Cells were incubated with DNA at 37°C for 20 min, diluted 1∶5 into sBHI, and incubated at 37°C for 80 min to allow expression of donor resistance alleles before dilution and plating to sBHI agar ± antibiotics [35]. Experiments were performed in triplicate from frozen aliquots of competent cultures prepared on three separate occasions. No DNA controls were performed in parallel, and antibiotic resistant colonies were not observed (limit of detection typically ∼10^−9^ resistant colonies/CFU). Cells from defrosted aliquots were pelleted and resuspended in fresh MIV before transformation. Two Nov^R^ and two Nal^R^ transformant colonies (Nov1, Nov2, Nal1, and Nal2; Table 1) were randomly selected for sequencing from a single experiment that used Rd-RR competent cells and NP-NN chromosomal DNA fragments (size range ∼20–100 kb).

### Whole-genome alignment of the reference sequences

The reference sequences for Rd and 86-028NP were compared using the Mauve whole-genome alignment software [42]. The complete genome sequences were aligned twice, once with Rd as the query and once with 86-028NP as the query. SNVs were then extracted using Mauve's “Export SNPs…” function. The few identified SNVs that were inconsistent between the two independent whole-genome alignments were excluded. The two resulting files provided positions of each SNV in each genome, ordered against one or the other reference.

### Illumina GA2 sequencing and initial data processing pipeline

Chromosomal DNA was sheared by nebulization, and converted into paired-end sequencing libraries with an insert size of ∼100–300 bp, as previously described [37]. About 10 million paired-end sequences of 42 bases were obtained from each library on individual lanes of an Illumina GA2 flow cell (Table S1). Raw data was processed using Illumina Pipeline Version 1.4, and all paired-end reads that passed standard Illumina quality control filters were used for analysis (*i.e.* those in the “.sequence.txt” file). The raw sequence reads for each DNA sample (Table S1) were deposited at the NCBI short-read archive under project accession SRP003474.

### Aligning sequence reads to the reference

The Rd (KW20) and 86-028NP complete genome sequences (NCBI genome accessions NC_000907 and NC_007416) were each used as references for read alignment, with the BWA algorithm (version 0.5.5) [40] set to highly sensitive alignment parameters (bwa aln -n 8 -o 3 -e 3 -l 20 -R 100000; bwa sampe -a 400 -o 1000000). While this generates some spurious mapping artifacts, it ensures that reads will map to both references when possible, even where there is high divergence.

### Processing the read alignments

A combination of two criteria was used to identify differences between sequence reads and their references and to flag positions with ambiguous base identity. The first method used the SamTools (version 1.12a)[41] consensus caller, which either assigns positions a standard A, C, G or T base or tags them as ambiguous. Reference positions missing from the SamTools consensus were treated as unmapped positions presumably within or near deletions. The second method used direct calculation of the frequency of each base at each reference position. This used a Perl script obtained from Galaxy [60] (pileup_parser.pl, parameter settings: 3 9 10 8 40 20 “No” “No” 2) to parse the pileup output from SamTools, and provided the count of each non-reference base call at each position. Parsed pileup files were subsequently analyzed using custom scripts written in the R statistical programming language [61]. Plots including gene maps were made with the assistance of the ‘genoPlotR’ package [62].

- **Control self-alignments.** Sequence differences between the Rd-RR and NP-NN strains and their respective reference sequences were identified using the following two criteria: (1) a difference was found by the SamTools consensus caller, and (2) the frequency of the same non-reference variant at that position was greater than 95%. Positions prone to sequencing and read mapping artifacts were flagged as ambiguous when the SamTools tagged a base as ambiguous, the variant frequency was between 5% and 95%, or both.
- **Control reciprocal alignments.** Differences between our donor and recipient strains were identified from the reciprocal alignments of Rd-RR reads to the 86-028NP reference genome and of NP-NN reads to the Rd reference genome. SNV positions were considered cross-validated, if both reciprocal alignments and whole-genome alignment identified the same SNV. Ambiguous positions prone to read mapping artifacts in reciprocal-alignments were also flagged using the same criteria as above.

### Identifying donor segments in individually sequenced transformed clones

Transformant sequence reads were analyzed as above. Recombination events were identified in the transformed clones by classifying the positions of cross-validated SNVs as donor, recipient, or ambiguous. Donor segments were defined as contiguous runs of donor-specific SNVs, uninterrupted by recipient-specific SNVs (ambiguous cross-validated SNV positions were ignored). Individual donor segments breakpoints were defined by the positions of their outermost donor-specific alleles. Donor segments were then manually inspected using the Integrated Genomics Viewer [63] to validate the donor segment breakpoint locations.

### Identifying donor segments in a pool of transformed clones

For the pooled sample of four transformed clones (RR3135-RR3138), donor-specific allele frequencies were determined at each cross-validated SNV position. Non-overlapping donor segments were unambiguously identified as contiguous runs of SNV positions with ∼25% donor-specific alleles. Overlapping donor segments (contiguous SNV positions with ∼50% donor-specific alleles) were disambiguated by comparison with the segments identified in RR3135 and RR3137. Segments unique to either RR3137 or RR3138 were disambiguated using allele-specific PCR; two primer pairs were designed that contained several SNVs that distinguished Rd and NP alleles (Table S13).

### Analysis of structural variation in and around donor segments

Positions that were unmapped by reads in the reciprocal alignments (but mapped in self-alignments) were used as markers of indel differences and other structural variation between donor and recipient, and the donor segment intervals were examined for read coverage at positions unmapped by either reciprocal alignment. Indel differences flanking the observed donor segments were also tabulated. Manual inspection of read alignments to both references used the Integrative Genome Viewer, and the “.rdiff” and “.qdiff” output from the dnadiff utility of Mummer [64] was used to cross-validate. GenomeMatcher [65] was used to view annotated sequence alignments at transforming and flanking structural variation to identify affected loci.

## Supporting Information

Figure S1 **Read depth varies consistently across the genome.** (**A**) shows a histogram of read depth per mapped Rd genome position. Red indicates Rd-RR, and blue indicates NP-NN. (**B**) shows a sliding window analysis (1 kb and 100 bp steps) of mean read depth of Rd-RR reads mapped to Rd (red) and %GC (grey) along an interval of the Rd genome. The genome-wide adjusted R^2^ of log(read depth) and %GC on these windows was 0.26 (using the lm function in R). (**C**) plots mean read depths on the same sliding windows as in **B**, but showing Rd-RR read depths on the x-axis and NP-NN read depths on the y-axis. The red line shows y = x. Unmapped positions were included as read depth  =  0, and mean read depths were adjusted by adding a single pseudo-count, so that read depths of 0 were plotted at 1 on a log-scale. (**D**) shows variation in read depth for Rd-RR reads mapped to the Rd reference genome along a representative interval. (**E**) shows the ratio of Nov1 to Rd-RR read depth along the same interval as (D). Read depths were first normalized to the median read depth to account for differences in sequence yields. The genome-wide correlation between read depths for these two samples was 0.98.
(TIF)

Figure S2 **Ambiguous positions.** Plot of the non-reference variant frequency at positions classified as ambiguous for the indicated set of sequence reads aligned to the two references: (**A**) Rd, and (**B**) 86-028NP. Data are tabulated in Table 2 and Table 3. The arrow indicates the 250 bp interval expanded in Figure S3. Note the high variant frequency of ambiguous positions at intervals in the two transformed clones at intervals containing donor segments when using the Rd reference genome (labeled with roman numerals as in Figure 2).
(TIF)

Figure S3 **Examples of two kinds of artifacts.** The 250 bps shown are indicated by the arrow in Figure S2B. (**A**) shows a systematic sequencing error-prone site, when either NP-NN or Rd-RR reads are mapped to the 86-028NP reference. (**B**) shows an additional problematic site, prone to systematic misalignment when Rd-RR reads are mapped to the 86-028NP genome, but not when NP-NN reads are mapped. The red curve shows the limit of detection (1/read depth). Grey bars show positions with no detected variants (i.e. variant frequency < limit of detection). Blue lollipops show the non-reference variant frequency at positions classified as matching the reference; when the lollipop falls on the limit-of-detection line, a single non-reference variant was observed. Turquoise bars show positions classified as ambiguous (variant frequency ranged from 12% to 65%). The salmon bars for Rd-RR reads indicate positions classified as SNVs. All were in the cross-validated set of SNVs, and the donor-specific allele frequency at each exceeded 98%.
(TIF)

Figure S4 **Density histograms of read depth per position (A and B) and non-reference variant frequency per position (C and D) when mapping control sequence reads to the 86-028NP reference.** **A** and **C** show the result using NP-NN reads, while **B** and **D** show reciprocal alignment of Rd-RR reads. Blue shows positions classified as a standard ACGT base by the SamTools consensus caller, while pink shows the histogram for positions classified as non-ACGT (AA and Aa, respectively). The percentages associated with each curve indicate the fraction of total positions in that group of positions (either ACGT or non-ACGT). Also shown in **B** and **D** is the percent of mapped positions where no non-reference variants were detected and the percent of unmapped positions.
(TIF)

Figure S5 **Unmapped positions in reciprocal alignments mark structural variation.** In (**A**), pink hatches mark positions along the Rd reference that were unmapped by NP-NN donor reads (but mapped by Rd-RR). In (**B**), light blue hatches mark positions along the 86-028NP reference that were unmapped by Rd-RR recipient reads (but mapped by NP-NN). Note the scale compresses the individual positions horizontally, so exaggerates the total fraction of unmapped positions (Table 2 and Table 3).
(TIF)

Figure S6 **False-positive positions.** Plots of non-reference variant frequency for each individually sequenced DNA sample at positions that were classified as “false positives”, SNVs found by alignment of donor reads to the Rd reference, but not identified as SNVs by whole-genome alignment (Figure 3, Step C). SNVs detected in self-alignments were first accounted for. Note the high variant frequency at “false positive” positions in the two transformant clones at intervals containing donor segments (labeled with roman numerals as in Figure 2).
(TIF)

Figure S7 **Zooms of the five intervals (I to V) containing donor-specific alleles in the transformants, as in** **Figure 4** **, plotted against the Rd reference genome.** The lower schematic shows each interval as in Figure 5B.
(TIF)

Figure S8 **Transformation of and near structural variation.** Shows Interval II for reads from the Nov1 clone mapped to the Rd reference (*top panel*) and also to the 86-028NP reference (*bottom panel*). In each plot, the top two rows show donor- and recipient-specific SNVs in blue and red, respectively. Light blue and pink bars that span the plot show donor- and recipient-specific structural variation, respectively. The purple diamond show the position of the Nov^R^ allele. The green line plots the log_2_ of Nov1 read depth normalized to either Rd-RR or NP-NN read depths (for the *top* and *bottom* panels, respectively). Positions where the green line touches the x-axis were unmapped by Nov1 reads.
(TIF)

Table S1 Summary of sequencing results.
(DOC)

Table S2 Read depth in pileups on Rd (KW20).
(DOC)

Table S3 Read depth in pileups on 86-028NP.
(DOC)

Table S4 Non-reference variants in reads mapped to Rd (KW20).
(DOC)

Table S5 Non-reference variants in reads mapped to 86-028NP.
(DOC)

Table S6 Summary of whole-genome alignment of Rd and 86-028NP reference sequences.
(DOC)

Table S7 Singe-nucleotide variants between Rd and 86-028NP reference sequences.
(DOC)

Table S8 Structural variation between Rd and 86-028NP reference sequences.
(DOC)

Table S9 Donor segments in four transformants using Rd (KW20) reference coordinates.
(DOC)

Table S10 Donor segments in four transformants using 86-028NP reference coordinates.
(DOC)

Table S11 Transformation of insertions and deletions within donor segments.
(DOC)

Table S12 Indel and other rearrangements flanking donor segments.
(DOC)

Table S13 Allele-specific primers used to assign Segments M, N, and O to either clone Nov2 or Nal2.
(DOC)
