# Supplementary figures and images for: Transformation of Natural Genetic Variation into Haemophilus Influenzae Genomes
Source: PLoS Pathog. 2011 Jul 28;7(7):e1002151. doi: 10.1371/journal.ppat.1002151 (PMC3145789; doi:10.1371/journal.ppat.1002151)

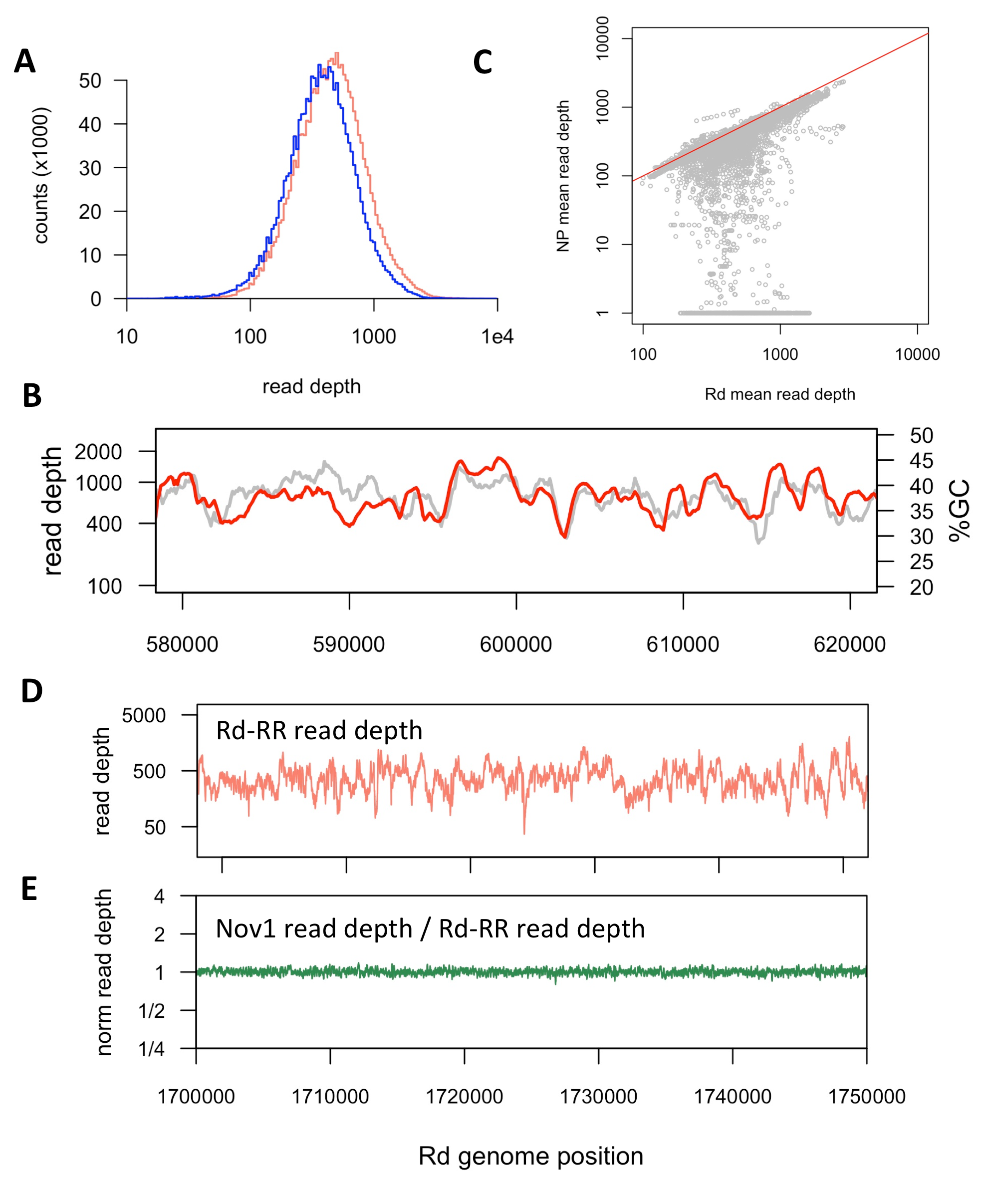

Supplement: Figure S1 — Read depth varies consistently across the genome. (A) shows a histogram of read depth per mapped Rd genome position. Red indicates Rd-RR, and blue indicates NP-NN. (B) shows a sliding window analysis (1 kb and 100 bp steps) of mean read depth of Rd-RR reads mapped to Rd (red) and %GC (grey) along an interval of the Rd genome. The genome-wide adjusted R2 of log(read depth) and %GC on these windows was 0.26 (using the lm function in R). (C) plots mean read depths on the same sliding windows as in B, but showing Rd-RR read depths on the x-axis and NP-NN read depths on the y-axis. The red line shows y = x. Unmapped positions were included as read depth = 0, and mean read depths were adjusted by adding a single pseudo-count, so that read depths of 0 were plotted at 1 on a log-scale. (D) shows variation in read depth for Rd-RR reads mapped to the Rd reference genome along a representative interval. (E) shows the ratio of Nov1 to Rd-RR read depth along the same interval as (D). Read depths were first normalized to the median read depth to account for differences in sequence yields. The genome-wide correlation between read depths for these two samples was 0.98. (TIF) [file ppat.1002151.s001.tif]

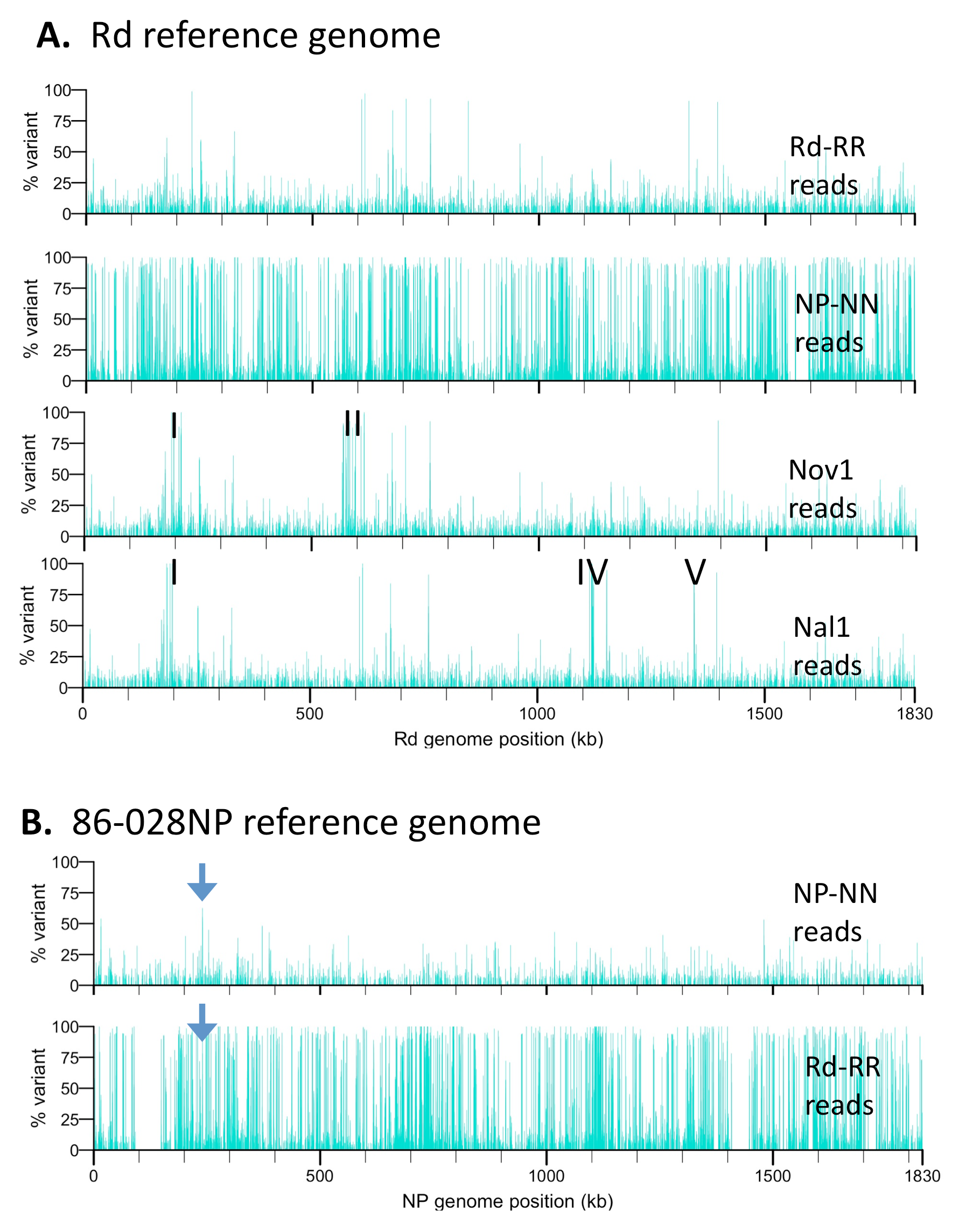

Supplement: Figure S2 — Ambiguous positions. Plot of the non-reference variant frequency at positions classified as ambiguous for the indicated set of sequence reads aligned to the two references: (A) Rd, and (B) 86-028NP. Data are tabulated in Table 2 and Table 3. The arrow indicates the 250 bp interval expanded in Figure S3. Note the high variant frequency of ambiguous positions at intervals in the two transformed clones at intervals containing donor segments when using the Rd reference genome (labeled with roman numerals as in Figure 2). (TIF) [file ppat.1002151.s002.tif]

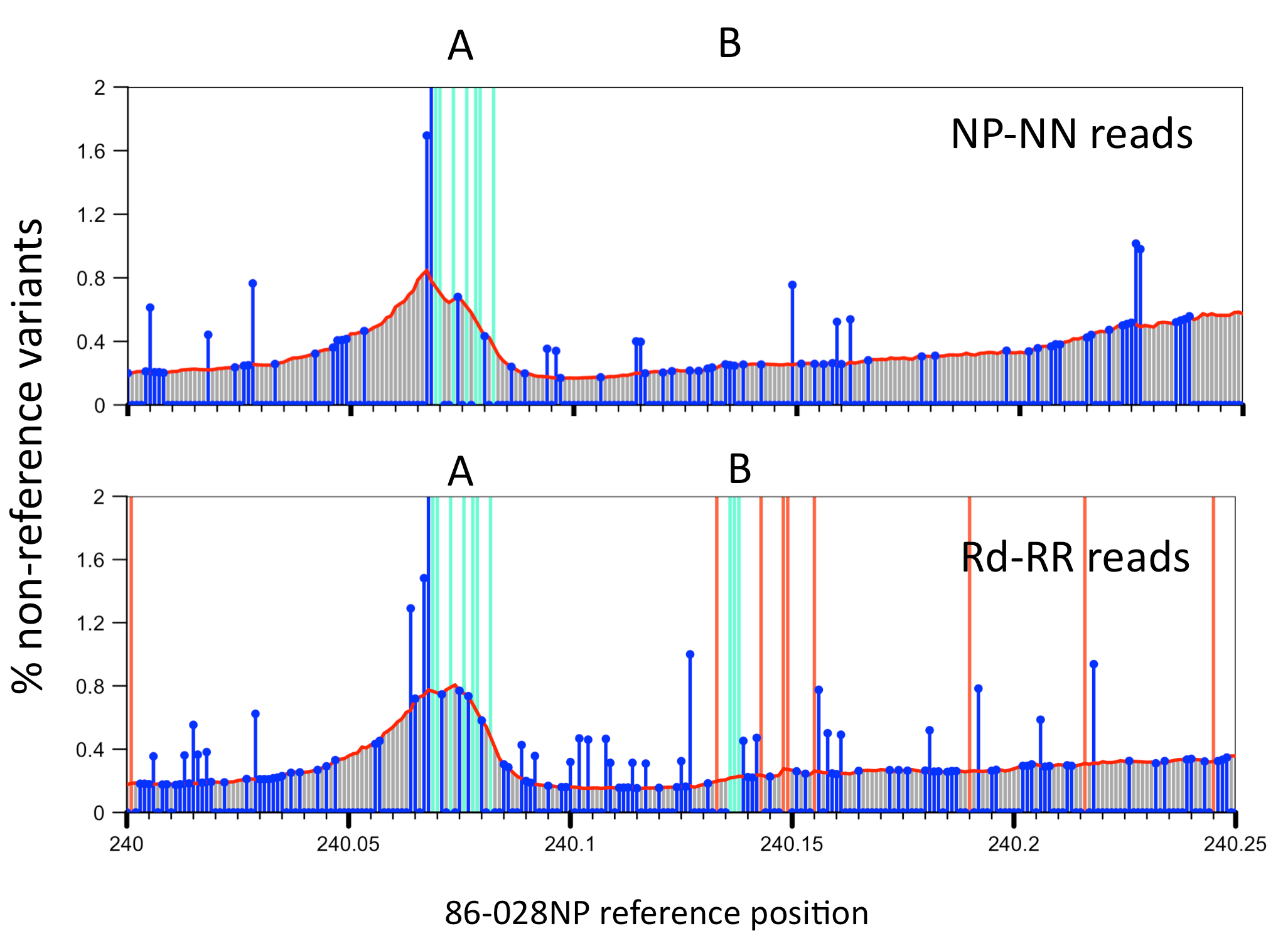

Supplement: Figure S3 — Examples of two kinds of artifacts. The 250 bps shown are indicated by the arrow in Figure S2B. (A) shows a systematic sequencing error-prone site, when either NP-NN or Rd-RR reads are mapped to the 86-028NP reference. (B) shows an additional problematic site, prone to systematic misalignment when Rd-RR reads are mapped to the 86-028NP genome, but not when NP-NN reads are mapped. The red curve shows the limit of detection (1/read depth). Grey bars show positions with no detected variants (i.e. variant frequency < limit of detection). Blue lollipops show the non-reference variant frequency at positions classified as matching the reference; when the lollipop falls on the limit-of-detection line, a single non-reference variant was observed. Turquoise bars show positions classified as ambiguous (variant frequency ranged from 12% to 65%). The salmon bars for Rd-RR reads indicate positions classified as SNVs. All were in the cross-validated set of SNVs, and the donor-specific allele frequency at each exceeded 98%. (TIF) [file ppat.1002151.s003.tif]

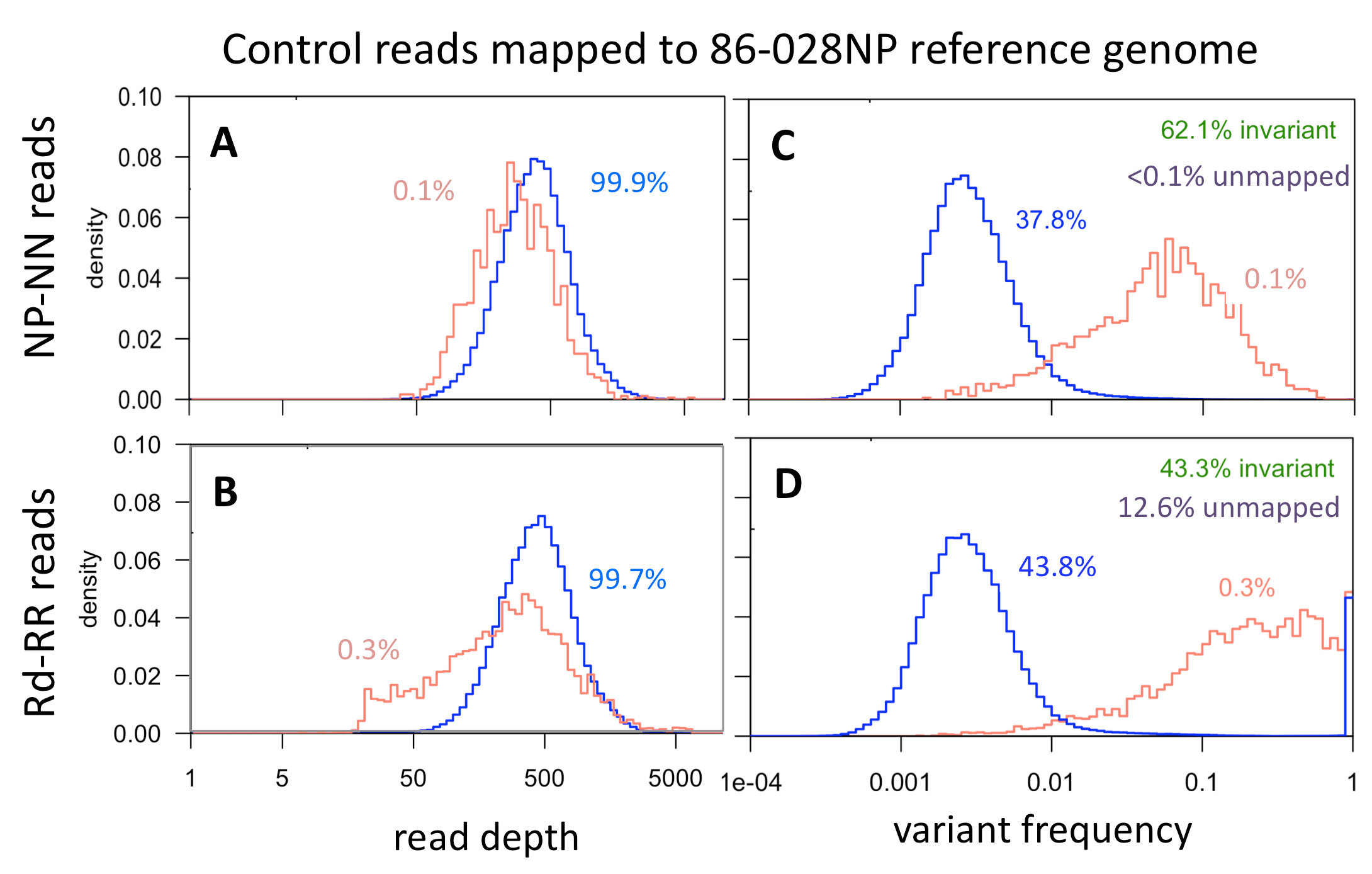

Supplement: Figure S4 — Density histograms of read depth per position (A and B) and non-reference variant frequency per position (C and D) when mapping control sequence reads to the 86-028NP reference. A and C show the result using NP-NN reads, while B and D show reciprocal alignment of Rd-RR reads. Blue shows positions classified as a standard ACGT base by the SamTools consensus caller, while pink shows the histogram for positions classified as non-ACGT (AA and Aa, respectively). The percentages associated with each curve indicate the fraction of total positions in that group of positions (either ACGT or non-ACGT). Also shown in B and D is the percent of mapped positions where no non-reference variants were detected and the percent of unmapped positions. (TIF) [file ppat.1002151.s004.tif]

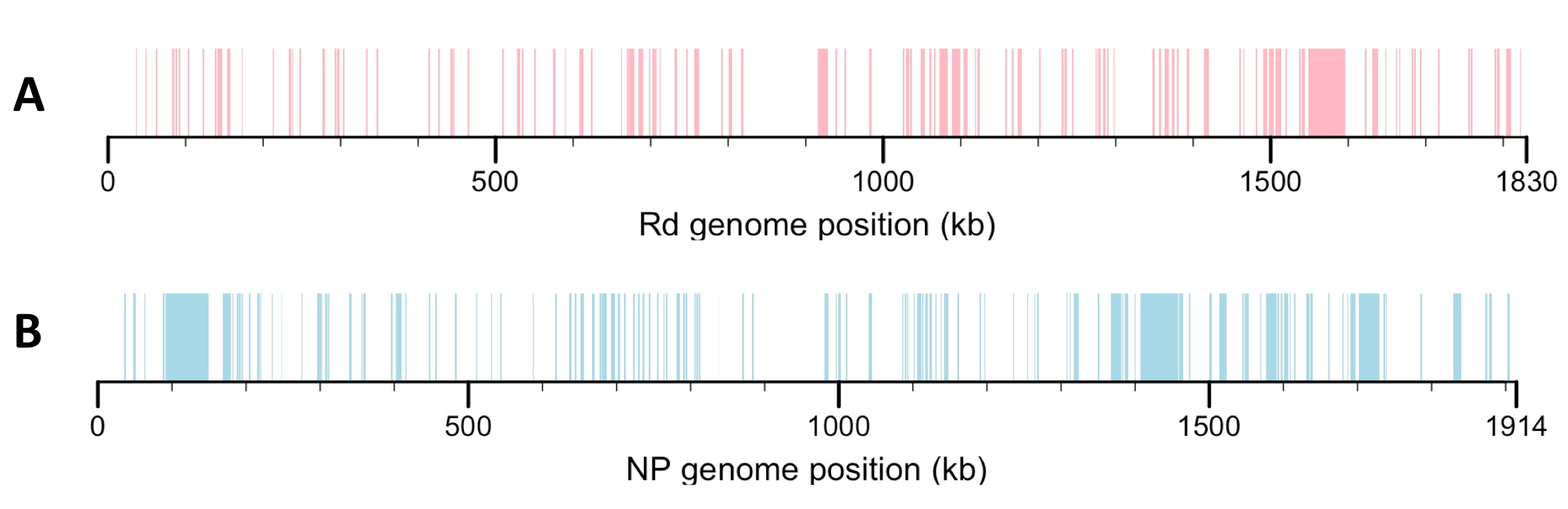

Supplement: Figure S5 — Unmapped positions in reciprocal alignments mark structural variation. In (A), pink hatches mark positions along the Rd reference that were unmapped by NP-NN donor reads (but mapped by Rd-RR). In (B), light blue hatches mark positions along the 86-028NP reference that were unmapped by Rd-RR recipient reads (but mapped by NP-NN). Note the scale compresses the individual positions horizontally, so exaggerates the total fraction of unmapped positions (Table 2 and Table 3). (TIF) [file ppat.1002151.s005.tif]

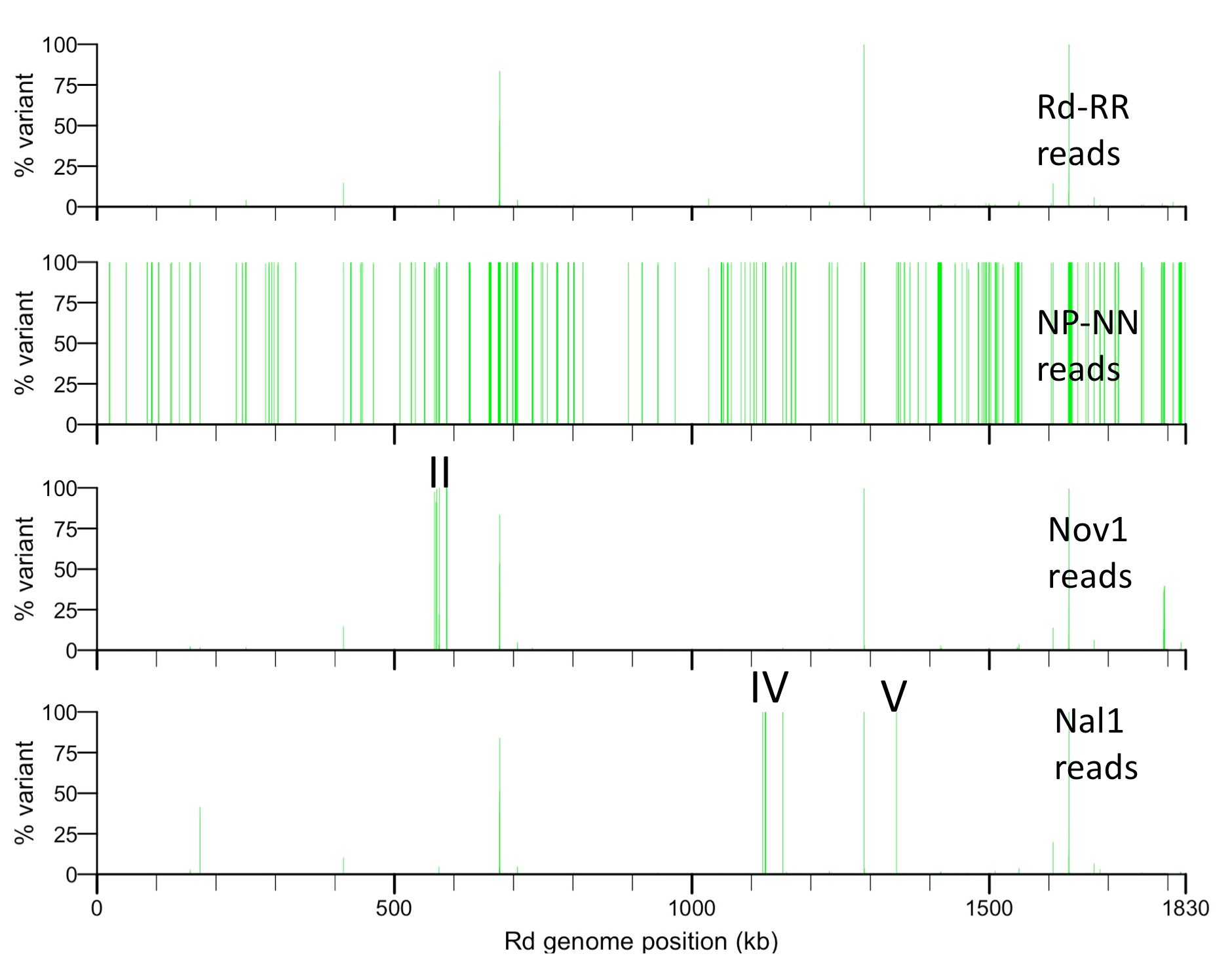

Supplement: Figure S6 — False-positive positions. Plots of non-reference variant frequency for each individually sequenced DNA sample at positions that were classified as “false positives”, SNVs found by alignment of donor reads to the Rd reference, but not identified as SNVs by whole-genome alignment (Figure 3, Step C). SNVs detected in self-alignments were first accounted for. Note the high variant frequency at “false positive” positions in the two transformant clones at intervals containing donor segments (labeled with roman numerals as in Figure 2). (TIF) [file ppat.1002151.s006.tif]

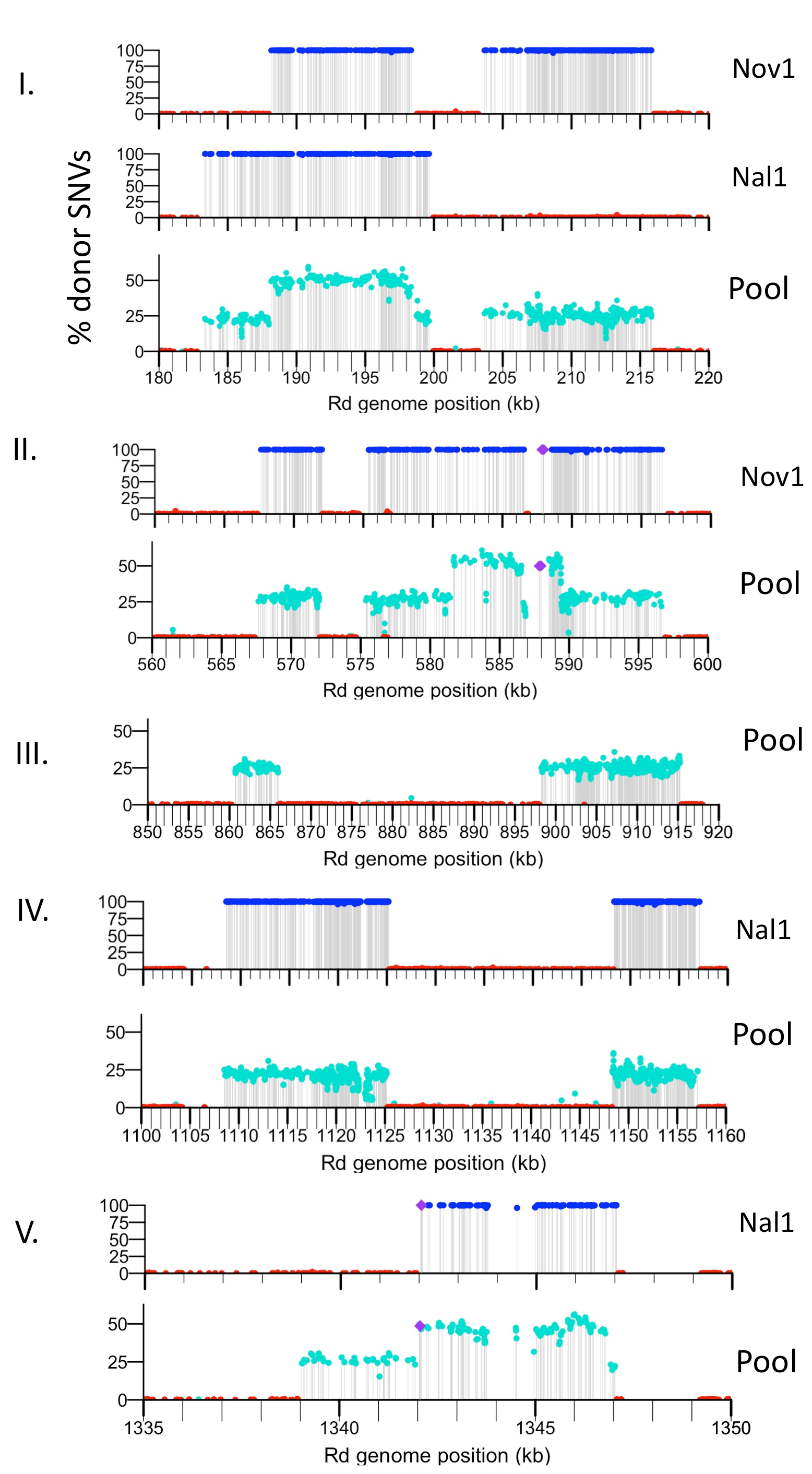

Supplement: Figure S7 — Zooms of the five intervals (I to V) containing donor-specific alleles in the transformants, as in Figure 4 , plotted against the Rd reference genome. The lower schematic shows each interval as in Figure 5B. (TIF) [file ppat.1002151.s007.tif]

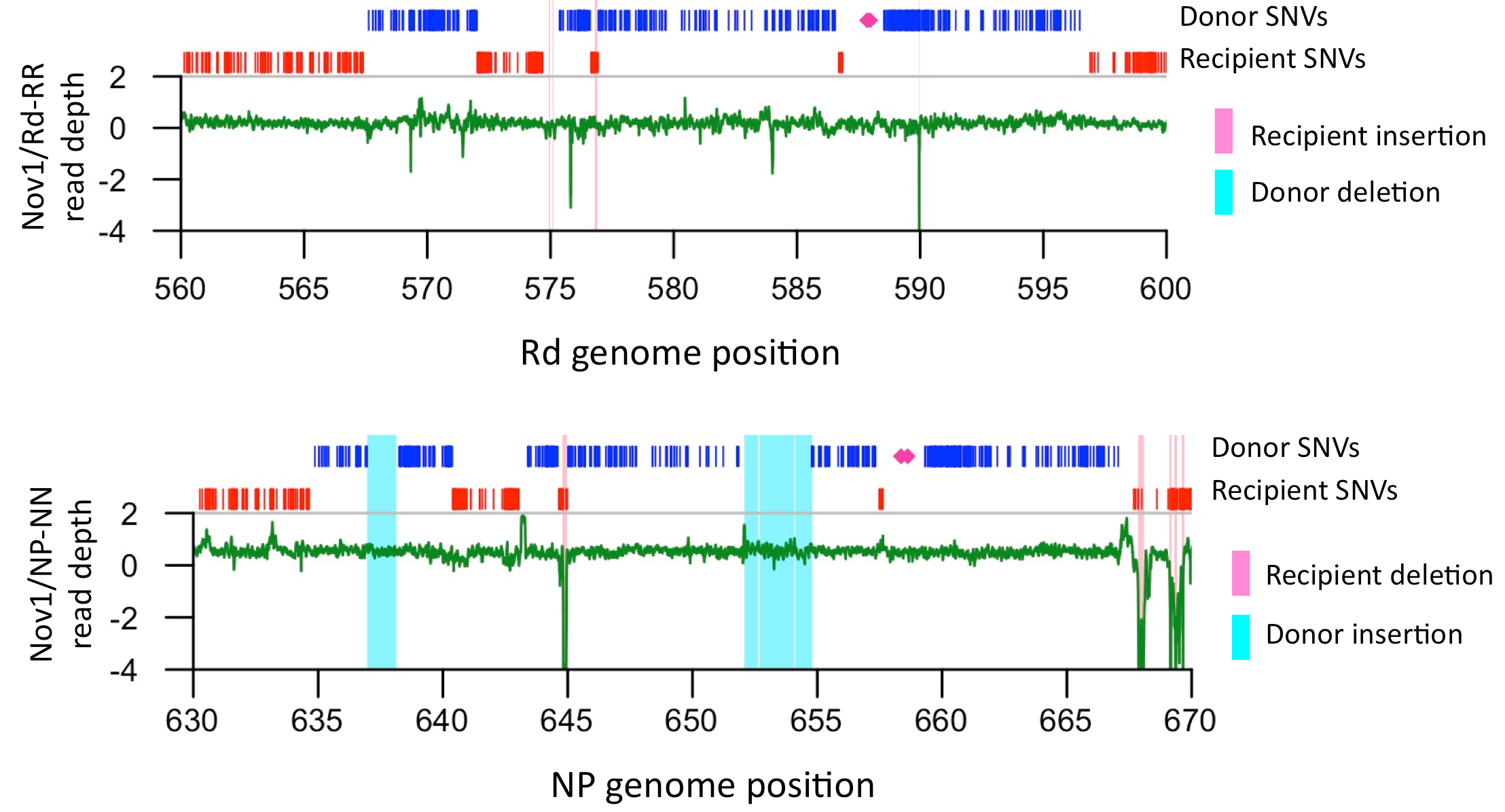

Supplement: Figure S8 — Transformation of and near structural variation. Shows Interval II for reads from the Nov1 clone mapped to the Rd reference (top panel) and also to the 86-028NP reference (bottom panel). In each plot, the top two rows show donor- and recipient-specific SNVs in blue and red, respectively. Light blue and pink bars that span the plot show donor- and recipient-specific structural variation, respectively. The purple diamond show the position of the NovR allele. The green line plots the log2 of Nov1 read depth normalized to either Rd-RR or NP-NN read depths (for the top and bottom panels, respectively). Positions where the green line touches the x-axis were unmapped by Nov1 reads. (TIF) [file ppat.1002151.s008.tif]
