# Supplementary material for: Transformation of Natural Genetic Variation into Haemophilus Influenzae Genomes
Source: PLoS Pathog. 2011 Jul 28;7(7):e1002151. doi: 10.1371/journal.ppat.1002151 (PMC3145789; doi:10.1371/journal.ppat.1002151)
Supplement: Table S1 — Summary of sequencing results. (DOC) [file ppat.1002151.s009.doc]

**Table S1: Summary of sequencing results**

|  |  |  |  | **% Unaligned Reads ^c^** | |
| --- | --- | --- | --- | --- | --- |
| **Lane** | **Sample** | **Read pairs ^a^** | **Mb ^b^** | **Rd (KW20) reference** | **86-028NP reference** |
| 1 | Rd-RR | 11,967,636 | 1,005.3 | 2.79% | 12.42% |
| 2 | NP-NN | 10,607,662 | 891.0 | 16.89% | 2.63% |
| 3 | Nov1 | 13,848,715 | 1,163.3 | 3.04% | 12.37% |
| 4 | Nal1 | 12,693,049 | 1,066.2 | 2.51% | 11.64% |
| 5 & 6 ^d^ | Pool | 26,347,570 | 2,213.2 | 3.13% | 12.10% |

a Total number of paired-end sequence reads passing the Illumina pipeline quality control

b Total megabases sequenced: 2 x 42 nt per paired-end read

c The % of reads that failed to map to the indicated reference using the BWA algorithm

d The pool consisted of equal amounts of genomic DNA from Nov1, Nov2, Nal1, and Nal2. Two lanes of Illumina GA2 sequencing were performed on the pool, and the two sequence datasets were merged using SamTools.
