# Supplementary material for: Transformation of Natural Genetic Variation into Haemophilus Influenzae Genomes
Source: PLoS Pathog. 2011 Jul 28;7(7):e1002151. doi: 10.1371/journal.ppat.1002151 (PMC3145789; doi:10.1371/journal.ppat.1002151)
Supplement: Table S2 — Read depth in pileups on Rd (KW20). (DOC) [file ppat.1002151.s010.doc]

**Table S2: Read depth in pileups on Rd (KW20)**

| **Lane** | **Sample** | **Median ^a^** | | **Low ^b^** | **High** |
| --- | --- | --- | --- | --- | --- |
| 1 | Rd-RR | 441 | ±261 | 18 | 6,048 |
| 2 | NP-NN | 369 | ±212 | *1 | 7,396 |
| 3 | Nov1 | 509 | ±301 | 10 | 6,606 |
| 4 | Nal1 | 531 | ±205 | 1 | 3,546 |
| 5 & 6 | Pool | 1,038 | ±529 | 16 | 8,745 |

a Median read depth per mapped position ± MAD (median absolute deviation)

b Lowest non-zero read depth at mapped positions. The * indicates that ≥ 1 position had zero read depth, despite having been mapped (likely an artifact of aligning reads with bases with systematically low quality scores).
