# Supplementary material for: Transformation of Natural Genetic Variation into Haemophilus Influenzae Genomes
Source: PLoS Pathog. 2011 Jul 28;7(7):e1002151. doi: 10.1371/journal.ppat.1002151 (PMC3145789; doi:10.1371/journal.ppat.1002151)
Supplement: Table S3 — Read depth in pileups on 86-028NP. (DOC) [file ppat.1002151.s011.doc]

**Table S3: Read depth in pileups on 86-028NP**

| **Lane** | **Sample** | **Median ^a^** | | **Low ^b^** | **High** |
| --- | --- | --- | --- | --- | --- |
| 1 | Rd-RR | 432 | ±258 | *1 | 11,174 |
| 2 | NP-NN | 380 | ±213 | 17 | 5,934 |
| 3 | Nov1 | 501 | ±298 | *1 | 12,378 |
| 4 | Nal1 | 524 | ±206 | *1 | 6,207 |
| 5 & 6 | Pool | 1,024 | ±526 | *1 | 16,527 |

a Median read depth per mapped position ± MAD (median absolute deviation)

b Lowest non-zero read depth at mapped positions. The * indicates that ≥ 1 position had zero read depth, despite having been mapped (likely an artifact of aligning reads with bases with systematically low quality scores).
