# Supplementary material for: Transformation of Natural Genetic Variation into Haemophilus Influenzae Genomes
Source: PLoS Pathog. 2011 Jul 28;7(7):e1002151. doi: 10.1371/journal.ppat.1002151 (PMC3145789; doi:10.1371/journal.ppat.1002151)
Supplement: Table S4 — Non-reference variants in reads mapped to Rd (KW20). (DOC) [file ppat.1002151.s012.doc]

**Table S4: Non-reference variants in reads mapped to Rd (KW20)**

| **Lane** | **Sample** | **Invariant ^a^** | **< 0.01 ^b^** | **< 0.05 ^b^** |
| --- | --- | --- | --- | --- |
| 1 | Rd-RR | 49.3% | 98.1% | 99.8% |
| 2 | NP-NN | 57.1% | 87.9% | 97.1% |
| 3 | Nov1 | 51.0% | 98.4% | 99.8% |
| 4 | Nal1 | 59.3% | 98.9% | 99.8% |
| 5 & 6 | Pool | 22.5% | 98.7% | 99.7% |

^a^ positions with no non-reference variants detected by mapped reads

^b^ positions with < 0.01 or <0.05 non-reference variants detected by mapped reads
