# Supplementary material for: Transformation of Natural Genetic Variation into Haemophilus Influenzae Genomes
Source: PLoS Pathog. 2011 Jul 28;7(7):e1002151. doi: 10.1371/journal.ppat.1002151 (PMC3145789; doi:10.1371/journal.ppat.1002151)
Supplement: Table S5 — Non-reference variants in reads mapped to 86-028NP. (DOC) [file ppat.1002151.s013.doc]

**Table S5: Non-reference variants in reads mapped to 86-028NP**

| **Lane** | **Sample** | **Invariant ^a^** | **< 0.01 ^b^** | **< 0.05 ^b^** |
| --- | --- | --- | --- | --- |
| 1 | Rd-RR | 43.3% | 83.3% | 97.1% |
| 2 | NP-NN | 62.1% | 98.6% | 99.9% |
| 3 | Nov1 | 45.2% | 83.9% | 97.2% |
| 4 | Nal1 | 51.7% | 84.1% | 97.2% |
| 5 & 6 | Pool | 21.0% | 84.2% | 97.1% |

^a^ positions with no non-reference variants detected by mapped reads

^b^ positions with < 0.01 or <0.05 non-reference variants detected by mapped reads
