# Supplementary material for: Transformation of Natural Genetic Variation into Haemophilus Influenzae Genomes
Source: PLoS Pathog. 2011 Jul 28;7(7):e1002151. doi: 10.1371/journal.ppat.1002151 (PMC3145789; doi:10.1371/journal.ppat.1002151)
Supplement: Table S6 — Summary of whole-genome alignment of Rd and 86-028NP reference sequences. (DOC) [file ppat.1002151.s014.doc]

**Table S6: Summary of whole-genome alignment of Rd and 86-028NP reference sequences**

|  | **total bps** |
| --- | --- |
| 86-028NP | 1,914,490 |
| KW20 | 1,830,138 |
| Unaligned | 434,453 |
| Identity in aligned segments | 97.66% |
