# Supplementary material for: Transformation of Natural Genetic Variation into Haemophilus Influenzae Genomes
Source: PLoS Pathog. 2011 Jul 28;7(7):e1002151. doi: 10.1371/journal.ppat.1002151 (PMC3145789; doi:10.1371/journal.ppat.1002151)
Supplement: Table S7 — Singe-nucleotide variants between Rd and 86-028NP reference sequences. (DOC) [file ppat.1002151.s015.doc]

**Table S7: Singe-nucleotide variants between Rd and 86-028NP reference sequences**

| **Class** | **bp** |
| --- | --- |
| Transitions | 24,339 |
| Transversions | 17,717 |
| Ambiguous ^a^ | 100 |
| **Total** | **42,156** |

^a^ Ambiguous bases are those with non-ACGT assignments in the KW20 reference genome
