# Supplementary material for: Transformation of Natural Genetic Variation into Haemophilus Influenzae Genomes
Source: PLoS Pathog. 2011 Jul 28;7(7):e1002151. doi: 10.1371/journal.ppat.1002151 (PMC3145789; doi:10.1371/journal.ppat.1002151)
Supplement: Table S8 — Structural variation between Rd and 86-028NP reference sequences. (DOC) [file ppat.1002151.s016.doc]

**Table S8: Structural variation between Rd and 86-028NP reference sequences**

| **Class ^a^** | **#** | **avg bp** | **total bp** |
| --- | --- | --- | --- |
| Insertions | 137 | 1,904 | 260,885 |
| Deletions | 149 | 1,162 | 173,199 |
| Relocations | 26 | 671 | 6,708 |
| Duplications | 22 | 923 | 20,315 |
| Inversion breakpoints | 20 |  |  |

^a^ Structural variant summary produced by the dnadiff utility of MUMmer. Insertions, deletions, and duplications are classified with respect to 86-028NP.
