# Supplementary material for: Transformation of Natural Genetic Variation into Haemophilus Influenzae Genomes
Source: PLoS Pathog. 2011 Jul 28;7(7):e1002151. doi: 10.1371/journal.ppat.1002151 (PMC3145789; doi:10.1371/journal.ppat.1002151)
Supplement: Table S9 — Donor segments in four transformants using Rd (KW20) reference coordinates. (DOC) [file ppat.1002151.s017.doc]

**Table S9**: Donor segments in four transformants using Rd (KW20) reference coordinates

| A | B | C | D | E | F | G | H | I | J | | K | |
| --- | --- | --- | --- | --- | --- | --- | --- | --- | --- | --- | --- | --- |
|  |  |  |  |  |  |  |  |  | **Nearest Rd SNV** | | **Putative Repair** | |
| **Clone** | **Seg** | **Int** | **Left Pos** | **Right Pos** | **SNVs** | **Length** | **Change** | **nt div** | **Left** | **Right** | **SNVs** | **Length** |
| Nov1 | A | I | 188,158 | 198,349 | 198 | 10,191 | 0 | 1.94% | 164 | 388 | 96 | 4,504 |
|  | B | I | 203,668 | 215,817 | 392 | 12,149 | 104 | 3.23% | 428 | 184 |  |  |
|  | C | II | 567,639 | 572,025 | 132 | 4,386 | 1,119 | 3.01% | 238 | 18 | 112 | 2,644 |
|  | D | II | 575,376 | 576,610 | 49 | 1,234 | -23 | 3.97% | *690 | 39 | 26 | *285 |
|  | E | II | 576,960 | 586,557 | 160 | 9,597 | 2,709 | 1.67% | 27 | 147 | 8 | 151 |
|  | F | II | 587,846 | 596,488 | *202 | 8,642 | 73 | 2.34% | *992 | *423 |  |  |
| Nal1 | G | I | 183,335 | 199,651 | 322 | 16,316 | -2 | 1.97% | 578 | 285 |  |  |
|  | H | IV | 1,108,531 | 1,125,162 | 452 | 16,631 | -153 | 2.72% | *2030 | 66 |  |  |
|  | I | IV | 1,148,347 | 1,157,094 | 336 | 8,747 | -21 | 3.84% | 58 | 129 |  |  |
|  | J | V | 1,342,043 | 1,347,039 | *108 | 4,996 | 17 | 2.14% | 45 | 111 |  |  |
| Nov2 | K | II | 581,677 | 583,795 | 14 | 2,118 | -1 | 0.66% | 243 | *226 | 4 | 10 |
|  | L | II | 584,044 | 589,428 | *117 | 5,384 | 4 | 2.17% | 14 | 21 |  |  |
|  | *M | III | 860,743 | 865,953 | 77 | 5,210 | 0 | 1.48% | 485 | 45 |  |  |
| Nal2 | *N | III | 898,259 | 903,482 | 99 | 5,223 | 0 | 1.90% | 247 | 25 | 1 | 1 |
|  | *O | III | 903,516 | 915,229 | 377 | 11,713 | 5 | 3.22% | 9 | 141 |  |  |
|  | P | V | 1,339,040 | 1,346,790 | *148 | 7,750 | 18 | 1.90% | 105 | 140 |  |  |

1. Clone containing indicated donor segment
2. Segment ID
3. Interval indicated in Figure 4A and used for plots in Figure S7. * indicates intervals whose coordinates are inverted relative to Rd (KW20)
4. Left-most donor-specific cross-validated SNV position defining the donor segment.
5. Right-most cross-validated donor-specific SNV position defining the donor segment.
6. Total cross-validated donor-specific SNVs detected in the segment. * denote a segment containing one of the two selected alleles conferring Nov^R^ or Nal^R^ resistance.
7. Length of donor segment, counting from the left- and right-most donor-specific SNVs.
8. Change in size (in bp) between donor segment length using 86-028NP reference coordinates and using Rd (KW20) reference coordinates (Table 5). A positive number indicates that transformation increased the length of the segment, and a negative indicates a decrease in length.
9. Nucleotide divergence between donor and recipient at alignable positions within the segment.
10. The distance to the nearest recipient-specific SNV position to the left and right of the donor segment coordinates. * indicate flanking distances that are disparate between using the Rd or 86-028NP reference coordinates by ≥10 bp (Figure S8).
11. Indicates the number of recipient-specific SNVs and minimal length contained in putative disruptions in longer recombination tracts that divide donor segments. The numbers refer to the recipient-specific gap between the indicated segment and the next line. * indicates a disparity between using Rd or 86-028NP reference coordinates by ≥10 bp.
