# Supplementary material for: Transformation of Natural Genetic Variation into Haemophilus Influenzae Genomes
Source: PLoS Pathog. 2011 Jul 28;7(7):e1002151. doi: 10.1371/journal.ppat.1002151 (PMC3145789; doi:10.1371/journal.ppat.1002151)
Supplement: Table S10 — Donor segments in four transformants using 86-028NP reference coordinates. (DOC) [file ppat.1002151.s018.doc]

**Table S10: Donor segments in four transformants using 86-028NP reference coordinates***

| A | B | C | D | E | F | G | H | I | J | | K | |
| --- | --- | --- | --- | --- | --- | --- | --- | --- | --- | --- | --- | --- |
|  |  |  |  |  |  |  |  |  | **Nearest Rd SNV** | | **Putative Gap** | |
| **Clone** | **Seg** | **Int** | **Left Pos** | **Right Pos** | **SNVs** | **Length** | **Change** | **nt div** | **Left** | **Right** | **SNVs** | **Length** |
| Nov1 | A | I | 250,187 | 260,378 | 198 | 10,191 | 0 | 1.94% | 164 | 386 | 96 | 4,502 |
|  | B | I | 265,694 | 277,947 | 392 | 12,253 | 104 | 3.20% | 429 | 184 |  |  |
|  | C | II | 634,880 | 640,385 | 132 | 5,505 | *1,119 | 2.40% | 229 | 18 | 112 | 2,644 |
|  | D | II | 643,406 | 644,617 | 49 | 1,211 | -23 | 4.05% | *360 | 39 | 26 | *344 |
|  | E | II | 645,026 | 657,332 | 160 | 12,306 | *2,709 | 1.30% | 27 | 147 | 8 | 151 |
|  | F | II | 658,354 | 667,069 | *202 | 8,715 | 73 | 2.32% | *725 | *620 |  |  |
| Nal1 | G | I | 245,364 | 261,678 | 322 | 16,314 | -2 | 1.97% | 578 | 285 |  |  |
|  | H | IV | 1,147,016 | 1,163,494 | 452 | 16,478 | -153 | 2.74% | *426 | 66 |  |  |
|  | I | IV | 1,186,685 | 1,195,411 | 336 | 8,726 | -21 | 3.85% | 58 | 129 |  |  |
|  | J | *V | 1,736,749 | 1,741,762 | *108 | 5,013 | 17 | 2.13% | 111 | 45 |  |  |
| Nov2 | K | II | 649,742 | 651,859 | 14 | 2,117 | -1 | 0.66% | 243 | *2932 | 4 | 10 |
|  | L | II | 654,814 | 660,202 | *117 | 5,388 | 4 | 2.17% | 14 | 21 |  |  |
|  | M | IV | 926,156 | 931,366 | 77 | 5,210 | 0 | 1.48% | 485 | 45 |  |  |
| Nal2 | N | IV | 963,648 | 968,871 | 99 | 5,223 | 0 | 1.90% | 247 | 25 | 1 | 1 |
|  | O | IV | 968,905 | 980,623 | 377 | 11,718 | 5 | 3.22% | 9 | 141 |  |  |
|  | P | *V | 1,736,998 | 1,744,766 | *148 | 7,768 | 18 | 1.89% | 140 | 105 |  |  |

* Columns headings as in Table S5
