# Supplementary material for: Transformation of Natural Genetic Variation into Haemophilus Influenzae Genomes
Source: PLoS Pathog. 2011 Jul 28;7(7):e1002151. doi: 10.1371/journal.ppat.1002151 (PMC3145789; doi:10.1371/journal.ppat.1002151)
Supplement: Table S11 — Transformation of insertions and deletions within donor segments. (DOC) [file ppat.1002151.s019.doc]

**Table S11: Transformation of insertions and deletions within donor segments**

| **Segment** | **Type (NP vs Rd)** | **Query** | **Left** | **Right** | **Description** |
| --- | --- | --- | --- | --- | --- |
| B | insertional deletion | Rd | 213,092 | 213,138 | 47 bp in *aroC-mepA* intergenic |
|  |  | NP | 275,135 | 275,268 | 134 bp in *aroC-mepA* intergenic |
| C | insertional deletion | Rd | 569,749 | 569,757 | 7 bp in infA-ksgA intergenic |
|  |  | NP | 636,991 | 638,146 | 1156 bp insertion of *lic2C* pseudogene (LPS biosynthesis) |
| E | insertion | Rd | 584,008 | 584,008 | 2706 bp deletion in *asnA-gph* intergenic |
|  |  | NP | 652,072 | 654,777 | 2706 bp insertion of 4 ORFs (putative transposon) |
| F | deletion | Rd | 591,068 | 591,069 | 2 bp insertion (AA) in *greB-HI0570* intergenic |
|  |  | NP | 661,842 | 661,842 | 2 bp deletion in *greB-HI0570* intergenic |
| H | insertional deletion | Rd | 1,122,336 | 1,123,042 | 708 bp in *HI1056* (putative type III metyltransferase) |
|  |  | NP | 1,160,829 | 1,161,490 | 663 bp in unannotated *HI1056* |
| I | deletion | Rd | 1,152,561 | 1,152,807 | 246 bp in *HI1087-sodA* integenic |
|  |  | NP | 1,190,899 | 1,191,120 | 221 bp in *HI1087-sodA* intergenic |
| J | insertional deletion | Rd | 1,344,618 | 1,344,965 | 347 bp in *gyrA-HI1265* intergenic |
|  |  | NP | 1,738,823 | 1,738,962 | 139 bp in *gyrA-HI1265* intergenic |
