# Supplementary material for: Transformation of Natural Genetic Variation into Haemophilus Influenzae Genomes
Source: PLoS Pathog. 2011 Jul 28;7(7):e1002151. doi: 10.1371/journal.ppat.1002151 (PMC3145789; doi:10.1371/journal.ppat.1002151)
Supplement: Table S12 — Indel and other rearrangements flanking donor segments. (DOC) [file ppat.1002151.s020.doc]

**Table S12**: Indel and other rearrangements flanking donor segments

| **Flanking** | **Type (NP vs Rd)** | **Query** | **Left** | **Right** | **Description** |
| --- | --- | --- | --- | --- | --- |
| C/D gap | deletion | Rd | 574,941 | 575,103 | 163 bp insertion in HI0554-HI0555 intergenic |
|  |  | NP | 643,299 | 643,135 | 163 bp deletion in HI0554-HI0555 intergenic |
| D/E gap | insertional deletion | Rd | 576,820 | 576,889 | 70 bp in *devB-zwf* intergenic |
|  |  | NP | 644,826 | 644,955 | 130 bp in *devB-zwf* intergenic |
| K/L gap | deletion | Rd | 584,008 | 584,008 | 2706 bp deletion in *asnA-gph* intergenic |
|  |  | NP | 652,072 | 654,777 | 2706 bp insertion of 4 ORFs (putative transposon) |
| F right | complex | Rd | 597,683 | NA | inversion breakpoint |
|  |  | NP | 667,183 | NA | inversion breakpoint |
| H left | insertional deletion | Rd | 1,104,214 | 1,108,427 | 4214 bp containing a type II R-M system |
|  |  | NP | 1,145,059 | 1,146,912 | 1854 bp containing conserved hypothetical ORF |
| I right | insertional deletion | Rd | 1,158,559 | 1,158,678 | 120 bp in *ccmF*-*dsbE* intergenic |
|  |  | NP | 1,196,876 | 1,196,999 | 124 bp in *ccmF*-*dsbE* intergenic |
| J left | insertional deletion | Rd | 1,347,191 | 1,349,197 | 2008 bp containing 3 conserved hypothetical ORFs |
|  |  | NP | 1,736,597 | 1,736,291 | 308 bp in *HI1266-HI1273* intergenic |
| O right | complex | Rd | 916,402 | 928,036 | 1 deletion and 1 insertion comprising 9 ORFs |
|  |  | NP | 981,796 | 985,605 | 2 insertions comprising 2 ORFs (*lic3A2* and *rmlB*) |
