# Supplementary material for: Transformation of Natural Genetic Variation into Haemophilus Influenzae Genomes
Source: PLoS Pathog. 2011 Jul 28;7(7):e1002151. doi: 10.1371/journal.ppat.1002151 (PMC3145789; doi:10.1371/journal.ppat.1002151)
Supplement: Table S13 — Allele-specific primers used to assign Segments M, N, and O to either clone Nov2 or Nal2. (DOC) [file ppat.1002151.s021.doc]

**Table S13: Allele-specific primers used to assign Segments M, N, and O to either clone Nov2 or Nal2**

| **Segment** | **Allele** | **Forward Primer** | **Reverse Primer** | **Size (bp)** |
| --- | --- | --- | --- | --- |
| M | Rd | C**G**ACTGCCACTTGATCGTT**A** | CAA**A**GCCTT**G**GT**G**AAAGT**C** | 224 |
|  | NP | C**T**ACTGCCACTTGATCGTT**G** | CAA**G**GCCTT**A**GT**A**AAAGT**G** | 224 |
| N | Rd | AATAGGCAATCCAACCAT**G**CC**A** | TGAT**T**GT**G**GAAGT**A**GGTGT**T**GG**A** | 295 |
|  | NP | AATAGGCAATCCAACCAT**A**CC**T** | TGAT**C**GT**A**GAAGT**C**GGTGT**A**GG**T** | 295 |
| O | Rd | GCA**AA**CA**T**GC**T**CCCAA**A** | AATTGAAGTCTTCGTACTTAAA**G**G**C** | 275 |
|  | NP | GCA**GG**CA**G**GC**A**CCCAA**G** | AATTGAAGTCTTCGTACTTAAA**A**G**A** | 275 |
